# Estimating the efficacy of Newborn-Communication, Health, Feeding and Swallowing Education Program (N-CHFSEP) for primiparous mothers

**DOI:** 10.12688/f1000research.152320.1

**Published:** 2024-07-09

**Authors:** Deepthi Ouseph, Jayashree Kanthila, Sunil Baliga, Shraddha Shetty, Sudhin Karuppali

**Affiliations:** 1 Department of Audiology and Speech Language Pathology, Kasturba Medical College Mangalore, Manipal Academy of Higher Education, Karnataka, Manipal, 576104, India; 2 Department of Pediatrics, Kasturba Medical College Mangalore, Manipal Academy of Higher Education, Karnataka, Manipal, 576104, India; 3 Department of Pediatrics, Yenepoya Medical College, Yenepoya University, Mangalore, Karnataka, 575018, India; 4 Department of Obstetrics and Gynecology, Kasturba Medical College Mangalore, Manipal Academy of Higher Education, Karnataka, Manipal, 576104, India

**Keywords:** accessible education, child health, early childhood development, health education, mental health, primiparous mothers

## Abstract

**Background:**

Primiparous mothers face diverse challenges during pregnancy and post-childbirth. There is a lack of comprehensive educational programs for primiparous mothers on maternal functioning and newborn care. This study aimed to explore the efficacy of a developed educational program on the attitude of primiparous mothers towards newborn communication, general health, feeding and swallowing. The objectives were (1) to develop an attitude questionnaire (AQ), a parent education program, and a feedback questionnaire (FQ); and (2) to estimate the efficacy of the education program pre- and post-delivery.

**Methods:**

Ninety-eight primiparous mothers without any obstetric history, proficient in English or Kannada, and delivering healthy newborns were recruited for the study. Phase 1 involved the development and validation of AQ, the parent education program [Newborn Communication, Health, Feeding and Swallowing Education Program (N-CHFSEP)], and FQ; while Phase 2 comprised of administering them on the mothers. Both quantitative (descriptive statistics, paired t-test, and chi-square test) and qualitative analysis were done on the parameters of interest.

**Results:**

The results of the study demonstrated a notable increase in the number of mothers (not all) reporting heightened confidence levels following receiving the N-CHFSEP (which was observed in all the domains). This observed change (pre and post) was statistically significant as per paired t-test analysis (p <0.05) indicating a significant increase in confidence levels post-N-CHFSEP intervention, as well as recognizing warning signs related to the same. Sociodemographic factors such as age, education, occupation, and family type were reported to have a significant effect (p <0.05) on maternal confidence levels before and after N-CHFSEP administration. Feedback from participants highlighted the effectiveness of the program in enhancing knowledge and awareness, while also suggesting areas for improvement.

**Conclusions:**

This study demonstrates the effectiveness of N-CHFSEP in enhancing primiparous mothers' confidence in newborn care, thereby improving maternal and infant health.

## Introduction

Pregnancy and childbirth represent a significant milestone in a woman's life permanently altering a woman's identity and way of living in a continuous, and dynamic manner. This phase is marked by significant physical and psychological transformations exerting a particular influence on primiparous mothers (first-time mothers) as they undergo a wide range of emotions including joy, excitement, and anxiety, along with the presence of overwhelming and stressful experiences such as routine newborn care, breastfeeding difficulties, lack of sleep, and physically taxing household duties. ^1^ Maternal confidence, knowledge, and attitudes play an important role during this critical period. ^2^ Reduced levels of confidence in primiparous compared to multiparous mothers negatively impact their ability to provide infant care. ^3^ The neonatal period becomes an important stage for brain development demanding a significant level of care from caregivers, facilitating the formation of new connections within the brain. ^4^ Developmental milestones (cognitive, gross, and fine motor, language, and social-emotional and behavioural) are a set of predetermined indicators that mark a child's achievement as they progress in their growth and development. The ability of caregivers to effectively identify these milestones facilitates early interventions, improving overall health outcomes. ^5^ Communicative skills are the best predictors in the development of school-age language, reading, and cognitive skills, helping in long-term development. Awareness of the development of early communication skills among primiparous mothers suggested the need for more reliable and approachable information about the development of communication skills. ^6^ Feeding and swallowing skills are considered promoters of physical, mental, and psychological health, providing infants with adequate nutrition required for development, which when unmet, may cause changes in the brain structure leading to general health challenges, cognitive deficits, and other metabolic, and behavioural difficulties. WHO (2010) has recommended certain guidelines for newborn care that includes maintaining cleanliness, ensuring adequate thermal protection, initiating proper breathing, providing eye care, promoting exclusive breastfeeding, administering immunizations, effectively managing illness, and offering specialized care for infants with low birth weight. Monitoring developmental milestones assumes a critical role in recognizing deviations and facilitating prompt interventions. ^5^

Maternal health literacy influences a woman's motivation and capacity to seek, comprehend, and apply information to enhance the well-being of both mother and child. ^7^ Such literacy programs provide newborn health indicators, especially useful for primiparous mothers as they encounter feelings of frustration and uncertainty, struggling to identify specific reasons for their infant’s issues. Consequently, most new postpartum mothers express a strong desire for professional guidance and support in both infant and self-care. ^1^ Studies on the needs of primiparous mothers have indicated their efforts to fulfil their informational requirements regarding pregnancy, through attending educational sessions and utilizing online resources to gather the necessary information. Numerous studies on primiparous mothers have highlighted the importance of educational programs delivered by healthcare professionals assisting in newborn care and the implementation of proper procedures to promote infant health. ^8^^,^^9^ A quasi-experimental study conducted on 72 primiparous mothers to understand the effect of the Maternal and Newborn Care Intervention (MNCI) program on maternal functioning revealed its efficacy in improving primiparous mothers functioning during the postpartum phase. ^10^ Similarly, Gozali and colleagues conducted a quasi-randomized controlled trial on 84 primiparous mothers in New York to evaluate the impact of a Newborn Education and Discharge Class, and they found mothers who took the programme to significantly exhibit higher levels of knowledge than the control group. ^3^ A pilot study conducted using a pre-/post-intervention design to assess the efficacy of the “HUG Your Baby” parent education and support program, indicated the parent education and support program fostered parent's general well-being, offering them the needed tools to ensure a seamless transition from the NICU to home, thereby providing them with confidence in their position as carers. ^11^ Several other universally used maternal literacy programmes have been reported to be used (1) the “Learn the Signs. Act Early.” (LTSAE) programme of the U.S. Department of Health and Human Services and the Centers for Disease Control and Prevention, ^12^ (2) the Mount Sinai Parenting Center (3), (3) the Internet Newborn-Care Education Programme (INCEP) developed in Taiwan, ^13^ (4) the postnatal psychoeducation programme developed in Singapore, ^14^ and (5) an educational support program developed in Iran. ^15^

The Early Childhood Development (ECD), a UNICEF global programme in India included schemes such as (1) the Home-Based Care for the Young Child (HBYC) developed by the National Health Mission as an expansion of the Home-Based Newborn Care (HBNC) programme, (2) the HBNC+ (an extension of HBNC), ^16^ (3) the Rashtriya Bal Swasthya Karyakram (RBSK), which is an initiative of the Government of India's Ministry of Health and Family Welfare, and (4) an updated version of the Mother and Child Protection (MCP) Card. ^17^ A cross-sectional study carried out on the use of MCP cards among 200 mothers with 1–12-month-old infants, indicated 86% of mothers to have read and comprehended the program, revealing the effectiveness of MCP cards as a health education tool for counselling during antenatal care visits in villages. ^18^ A systematic review and meta-analysis were carried out to understand the effectiveness of educational and support intervention program on breastfeeding rates among primiparous women at six months and up to two years of postpartum period. The results indicated that the rate of breastfeeding in the intervention group at six months was twice as high as in the control group when the intervention combined both antenatal and postnatal teaching and support. ^19^ Although these initiatives have covered a range of aspects of mother and child development, there exists a lack of a holistic educational programme covering important areas of baby development, such as feeding and swallowing, communication, and newborn health. This emphasises the need for a comprehensive educational module addressing a range of important developmental aspects for mothers with their newborns. Access to such reliable sources of information empowers primiparous women to enhance their confidence and shape their mental expectations and ideals regarding pregnancy and childbirth. Additionally, such programs would enable them to feel more assured and better formulate mental expectations and principles concerning their pregnancy and childbirth experiences. ^20^ There are only a handful of centres that mandatorily provides such information to primiparous mothers with a dearth of existing studies in India pertaining to the efficacy of using such education programs. With primiparous mothers showing reduced levels of maternal confidence than multiparous mothers towards providing infant care (3), the current study aimed to explore the efficacy of an educational program on the attitude of primi mothers towards milestones of early infant communication, feeding-swallowing and general health of their infants. The objectives of the study were (1) to develop and validate an attitude questionnaire, parent education program and feedback questionnaire; (2) and to estimate the efficacy of an education program pre and post-intervention delivery.

## Methods

The research was a single-arm pre-post study carried out between August 2023 and February 2024. A non-random convenience sampling method was adopted for the study. The study was approved by the Institutional Ethics Committee (IEC KMC MLR 03/2023/108) of Kasturba Medical College, Mangalore, Manipal Academy of Higher Education on 20.07.2023 and was registered under the Clinical Trials Registry of India (CTRI/2023/05/053109) on 25.05.2023. We used the CONSORT checklist when writing our report. ^21^ The study has adhered to the principles of Declaration of Helsinki.

### Participants

The sample size was calculated using the formula: n= [Z_(1-α/2)^2×pq]/d^2; where Z = 1.96, standard normal at 5% level of significance; p = 0.47%, q= 1-p, d=0.1. ^22^ A total of 98 primiparous mothers [mean age (in years): 26.03 ±3.79] were recruited for the study. Seventy-six (77.6%) of them belonged to a joint family, while 22 (22.4%) were from a nuclear family. These mothers comprised homemakers (62%), homemakers after having resigned from their jobs due to pregnancy (11%), working in educational sectors (8%), and other private/government sectors (19%). The educational qualifications indicated mothers have completed their basic formal education (38%), higher secondary education (19%), diploma (35%), graduation (35%), and postgraduation (3%).

The inclusion criteria included (1) inpatient primiparous mothers without any prior obstetric history, (2) mothers having given birth to healthy newborns through various delivery methods [vaginal, assisted vaginal (including vacuum, forceps, or cesarean)], and (3) mothers proficient in English or Kannada (a South Indian language spoken in the state of Karnataka). The exclusion criteria included (1) primiparous mothers delivering twins/triplets with significant history of obstetric, medical, or psychological problems, and (2) mothers who were health professionals. Multiparous mothers were excluded due to their experience in caring for newborns. Mothers with obstetric complications were excluded as that may affect their ability to participate in the educational program or effectively express their attitudes. All participants were recruited from Government Lady Goschen Hospital (a government hospital exclusive for maternity care), and Kasturba Medical College Hospital (a multidisciplinary speciality centre) of the Mangalore taluk, Dakshina Kannada district.

### Procedure

The present study was conducted in 2 phases. Phase 1 included the development of an (a) attitude questionnaire, (b) parent education program, and (3) feedback questionnaire; while Phase 2 included the administration of the questionnaires and the education program on the participants, followed by data analysis of the retrieved data.

Phase 1: Development of the tools.

a) Attitude questionnaire (AQ): The AQ was developed by reviewing relevant literature ^8^^,^^21^^–^^25^ on primiparous mothers' attitudes towards infant communication, feeding-swallowing practices, and general health. The questionnaire was designed to encompass various domains (communication milestones, feeding schedules, warning signs during feeding-swallowing and milestones, newborn hygiene, bathing care, vitamin supplements, immunizations, developmental milestones, and general care). The final version of the questionnaire (Appendix-I) comprised 16 closed-ended statements, each designed to assess maternal confidence in the above-mentioned domains. Items 1, 2, 3, 5, 7 were pertaining to communication skills and represented as C1, C2, C3, C5, and C7 respectively. Items 4, 6, 8, 9 and 12 were related to feeding-swallowing skills indicated as F4, F6, F8, F9 and F12 respectively. Finally, items 10, 11, 13, 14, 15 and 16 focused on newborn health and were indicated as H10, H11, H13, H14, H15 and H16 respectively. Each item was to be rated using a 5-point Likert rating scale (ranging from 5 being extremely confident to 1 being extremely unconfident). Additionally, the questionnaire included three open-ended questions indicated as G17, G18 and G19 targeting the mother's attitudes, concerns and opinions on the importance of communication and the well-being of newborns.
b) Parent Education Program: The parent education program [Newborn-Communication, Health, Feeding and Swallowing Education Program (N-CHFSEP)] was developed after reviewing the existing literature ^12^ on the developmental milestones of newborns. This provided an initial framework for the education program which was further developed with discussions with experienced pediatricians and speech-language pathologists (SLPs). The program covered essential information incorporating a comprehensive set of monthly milestones (0-6 months) crucial for development. The N-CHFSEP featured a total of 120 statements, each appropriately categorized within the corresponding age range. The following table ( Table 1) depicts the general framework of the N-CHFSEP used in the current study.
c) Feedback questionnaire (FQ): The FQ was developed after reviewing the literature ^13^^,^^19^^,^^26^^,^^27^ on first-time mother's feedback towards parent education on infant communication, feeding-swallowing and general health of infants. A conceptual framework was developed which was expanded based on discussions with experienced pediatricians and SLPs. This questionnaire (Appendix-II) contained five closed-ended statements following a 5-point Likert rating scale (ranging from 5 being strongly agree to 1 being strongly disagree). Additionally, there was one open-ended question, allowing mothers to share their suggestions regarding the N-CHFSEP.

**Table 1.** General framework of the N-CHFSEP developed for the study.

Age range (months)	0-1	1-2	2-3	3-4	4-5	5-6
Statements	20	19	19	21	16	20
Age wise suggestions	3	1	0	1	0	0
Total items	23	20	19	22	16	20
General suggestions	12					
Red flags	21					

Content Validation

The AQ, FQ, and N-CHFSEP were subjected to content validation by an expert panel to evaluate their adherence to established scientific facts and the grammatical accuracy of the designed statements. The panel included three primiparous mothers who were professionally qualified speech-language pathologists (SLPs) and three primiparous mothers who were professionally qualified paediatricians, each of them having a minimum of five years of clinical experience. All experts received a copy of the developed questionnaires (AQ and FQ) and N-CHFSEP. Each expert reviewed the tools (item-wise) on a 3-point Likert rating scale (0 being inappropriate, 1 being modifications required, and 2 being appropriate). For the N-CHFSEP validation, the panel evaluated the suitability of the milestones to appear under the corresponding age (0-6 months). Following the content validation by SLPs and the pediatricians, the content validation index (CVI) was calculated at both the item and scale level for the AQ, N-CHFSEP, and FQ respectively. The Item level CVI (I-CVI) was computed by dividing the number of experts who rated the items as one or two by the total number of experts. Scale level CVI (S-CVI) was then calculated by averaging the I-CVI scores for AQ, and FQ. The resulting S-CVI scores were 0.95 for AQ, 0.90 for N-CHFSEP, and 1.0 for FQ. The I-CVI scores for the N-CHFSEP fell below the desired level (0.78), indicating insufficient agreement among experts and, hence demanding a revalidation. The same expert panel participated in this process to obtain an I-CVI score of 1.

The AQ, FQ and N-CHFSEP underwent grammatical modifications (e.g. ‘The child … ….’ to ‘My baby … ….’) to enhance their comprehensibility for mothers. Based on expert advice, the N-CHFSEP was revised to highlight potential warning signs using the checklist released by the Centers for Disease Control and Prevention (CDC), ^12^ within each month and across the 6 months. Furthermore, the program was revised to incorporate recommendations for fostering the child's communication abilities and holistic growth. Specific suggestions were made to include illustrations of certain elements in the N-CHFSEP, to facilitate better comprehension of the presented content. The SLPs provided valuable input, including guidance on breastfeeding practices, as well as identifying red flags related to communication and feeding milestones. The pediatric experts suggested the reorganization of milestones by months and repeating specific items in their respective months. Additionally, it was recommended to develop a pamphlet on the N-CHFSEP which would serve as a carry-home resource for mothers. Following the incorporations of the modifications the final N-CHFSEP was ready for administration.

Translation

The AQ, N-CHFSEP, and FQ were translated into Kannada (one of the common regional languages spoken in the Mangalore taluk). The AQ, N-CHFSEP, and FQ underwent both forward and backward translations which were performed by a bilingual translator proficient in both Kannada and English, with expertise in the subject matter. Following the completion of both forward and backward translations, a final Kannada version of the AQ, N-CHFSEP, and FQ was ready for administration. A pilot study was conducted on two Kannada-speaking participants to assess the comprehensibility, clarity, and cultural relevance of the finalized translated AQ, N-CHFSEP, and FQ. This continuous process of forward and backward translation contributed significantly to the questionnaire's accuracy and cultural appropriateness.

Phase 2: Administration of the tools and data analysis

The data collection took place in the postnatal ward through one-on-one interactions. Participants were recruited by reviewing their medical records based on the inclusion and exclusion criteria. The data was collected from each of the participants on the second day following their delivery due to the discomfort commonly experienced by the mothers on the first-day post-delivery. Safety and hygiene were strictly adhered to by following hand hygiene protocols and by wearing surgical masks (as per WHO guidelines) before approaching the participants at their bedside. Efforts were made to facilitate participants to be seated in a comfortable position, enhancing effective interaction. The researcher obtained written informed consent from the participants followed by their socio-demographic details. Each participant was oriented to the study goals and objectives and was provided with comprehensive information pertaining to the questionnaires and the education program they will be subjected to. They were actively encouraged to seek clarifications on any uncertainties pertaining to the items within the questionnaire. The study commenced by providing each participant with the AQ which required them to read the questions and mark their level of confidence across each item, followed by the presentation of the N-CHFSEP, during which the examiner verbally presented the participants with the program, further to which a printed version of the N-CHFSEP was provided, after which the AQ was re-administered. Subsequently, feedback regarding the N-CHFSEP was obtained from the participants using the FQ. All verbal responses from the mothers were recorded using a digital voice recorder (Sony ICD-UX560F Stereo IC recorder 4 GB). The total administration spanned a duration of 25-30 minutes.

The digital voice recordings of the primiparous mothers' responses towards the AQ and FQ administration were transcribed and subjected to quantitative analysis. All open-ended questions were subjected to qualitative analysis by categorizing the verbatim into comprehensive themes to examine the responses received. Descriptive statistics (quantitative data) were performed using SPSS version 25.0. The continuous variables were analyzed using mean and standard deviation while the discrete variables were analyzed using frequency and percentage. The efficacy of the N-CHFSEP was estimated by comparing the pre and post-AQ scores and analyzing the FQ responses. A paired sample t-test was performed to determine the confidence levels (before and after intervention) of primiparous mothers across domains of communication skills, feeding-swallowing skills and general newborn health. Chi-square tests were carried out to determine the relationship between the demographics of the primiparous mothers and their confidence levels under each domain (communication skills, feeding -swallowing skills and general newborn health).

## Results

**Figure 1.**
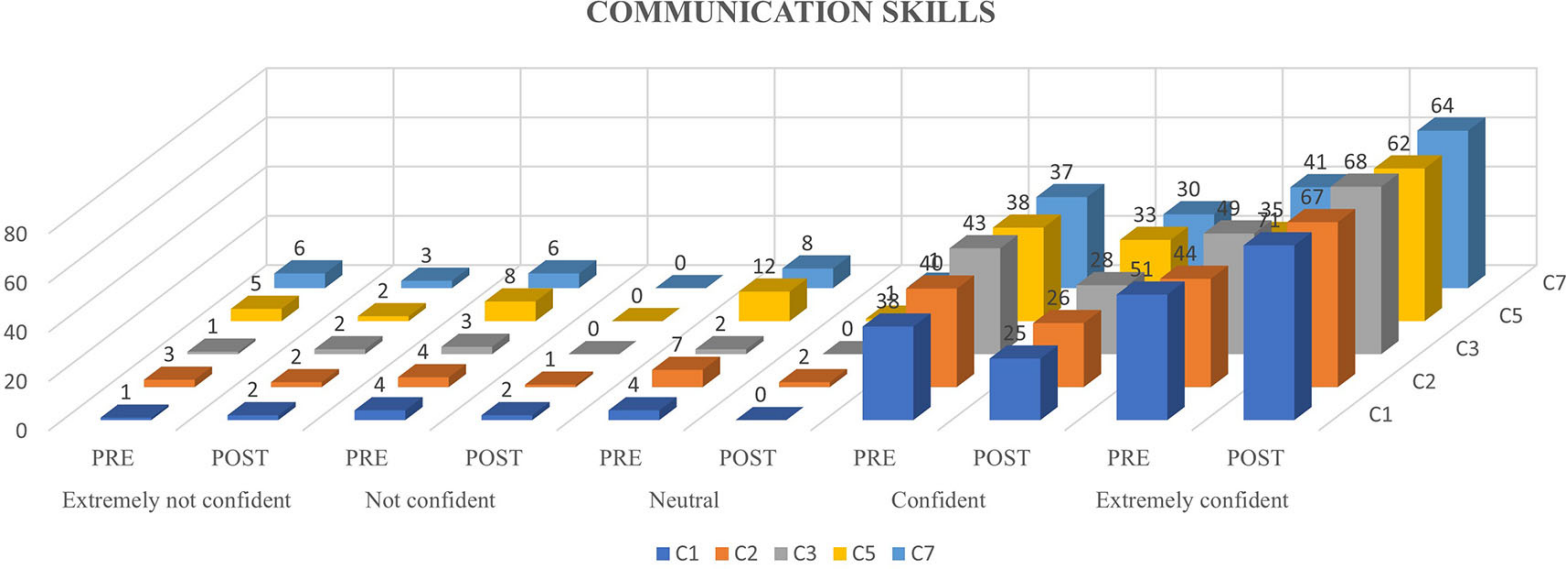
The confidence levels (pre and post administration of the PEP) of primiparous mothers in estimating the communication skills of the newborn.

**Figure 2.**
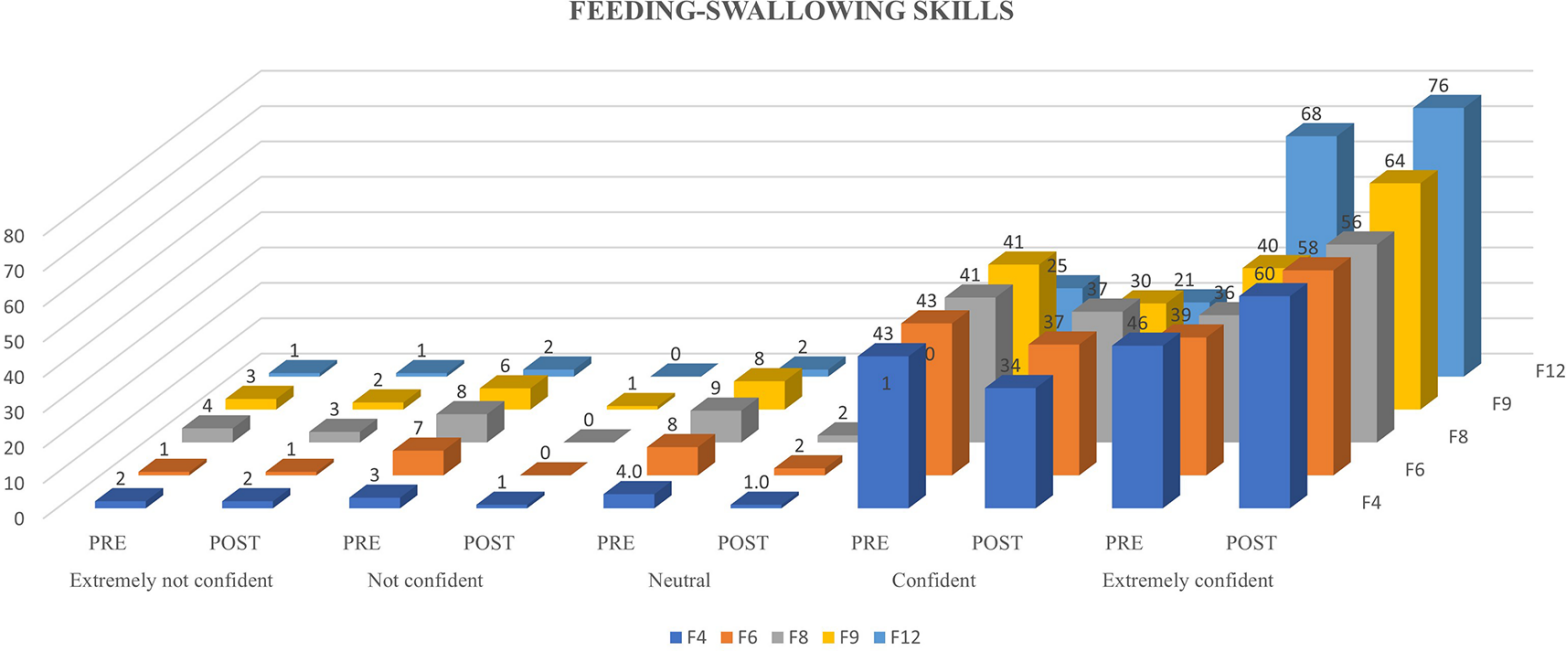
The confidence levels (pre and post administration of the PEP) of primiparous mothers in estimating the feeding-swallowing skills of the newborn.

**Figure 3.**
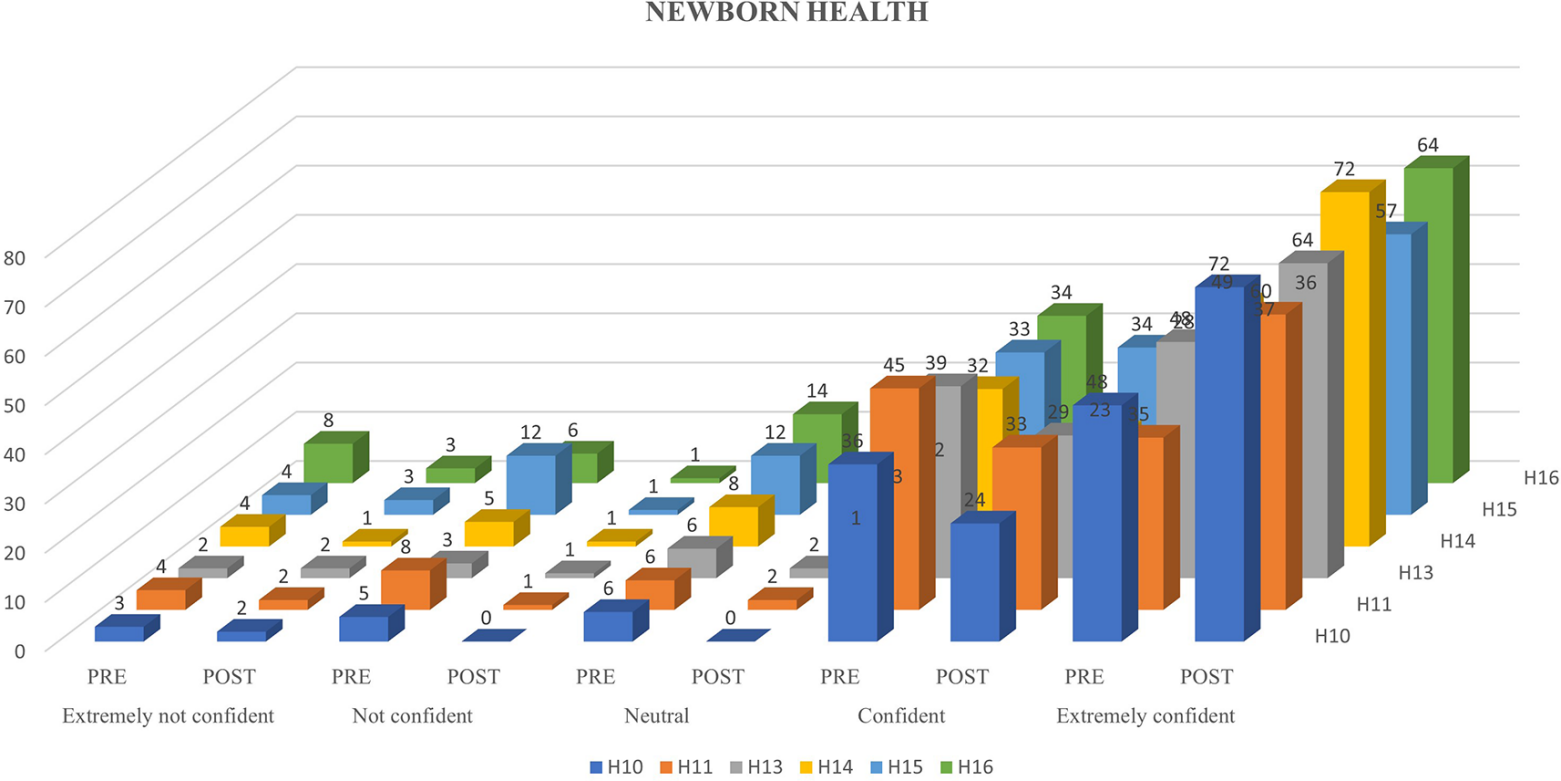
The confidence levels (pre and post administration of the PEP) of primiparous mothers in estimating the health concerns of the newborn.

Ninety-eight participants were involved in the study and underwent the N-CHFSEP. Figures 1, 2 and 3 represents the confidence levels (before and after administration of PEP) of primiparous mothers in judging the communication skills, feeding-swallowing skills, and general health concerns of their infants respectively.

**Figure 4.**
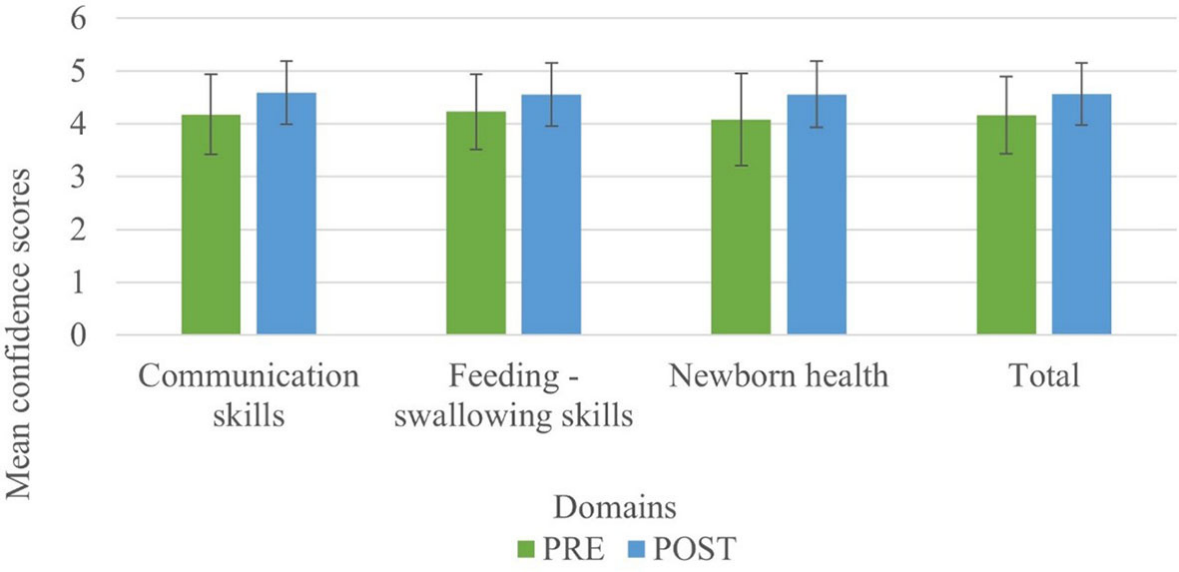
The mean (SD) confidence levels of primiparous mothers before and after the administration of PEP.

Figure 4 represents the before-after confidence levels of primiparous mothers in judging communication, feeding-swallowing, and health concerns of infants.

### Comparison of confidence levels of primiparous mothers following administration of the N-CHFSEP

Table 2 represents the results of a paired sample t-test conducted to compare the effect of N-CHFSEP on confidence levels of primiparous mothers.

**Table 2.** Comparison of total scores of pretest and post-test confidence levels.

Sl. No	Domain	Mean Differences	Std. Deviation	t	Sig. (2-tailed)
1	Communication skills	0.41633	0.69084	5.966	0.000
2	Feeding- swallowing skills	0.33469	0.63361	5.229	0.000
3	Newborn health	0.47959	0.70437	6.740	0.000
	Total Pre-Post	0.41020	0.61271	6.628	0.000

### Effect of age, education, family type, and occupation on the confidence levels reported by primiparous mothers before and after the administration of the N-CHFSEP

The chi-square correlation values between age, education, family type, and occupation towards the total pre-post-test measures of communication skills, feeding-swallowing skills and general infant health are represented in Table 3.

**Table 3.** The pre-post-test chi-square correlation values between participant demographics, and communication, feeding-swallowing, and general health.

Demographics	Chi-square correlation value		
	Communication skills	Feeding – swallowing skills	General infant health
Age			
18-25	χ ^2^ (77) =103.345, p=0.024 *	χ ^2^ (66) =106.669, p=0.001 *	χ ^2^ (105) =177.160, p=0.000 *
26-35	χ ^2^ (104) =240.433, p=0.000 *	χ ^2^ (96) =155.498, p=0.000 *	χ ^2^ (192) =383.548, p=0.000 *
Education			
Degree	χ ^2^ (77) =157.691, p=0.000 *	χ ^2^ (54) =69.190, p=0.080	χ ^2^ (99) =148.575, p=0.001 *
Post degree	χ ^2^ (2) =3.000, p=0.223	χ ^2^ (2) =3.00, p=0.223	---
<=10th STD	χ ^2^ (54) =89.901, p=0.002 *	χ ^2^ (50) =79.001, p=0.006 *	χ ^2^ (91) =158.504, p=0.000 *
<=12th STD	χ ^2^ (40) =61.394, p=0.016 *	χ ^2^ (42) =52.883, p=0.121	χ ^2^ (66) =87.083, p=0.042 *
Diploma	χ ^2^ (8) =10.000, p=0.265	χ ^2^ (12) =15.000, p=0.241	χ ^2^ (12) =15.000, p=0.241
Family Type			
Joint Family	χ ^2^ (104) =235.627, p=0.000 *	χ ^2^ (96) =208.574, p=0.000 *	χ ^2^ (216) =470.522, p=0.000 *
Nuclear Family	χ ^2^ (35) =42.472, p=0.180	χ ^2^ (45) =51.897, p=0.223	χ ^2^ (48) =74.000, p=0.009 *
Occupation			
Homemaker	χ ^2^ (98) =180.349, p=0.000 *	χ ^2^ (91) =170.998, p=0.000 *	χ ^2^ (180) =344.503, p=0.000 *
Homemaker resigned	χ ^2^ (40) =48.889, p=0.158	χ ^2^ (15) =20.854, p=0.142	χ ^2^ (36) =44.000, p=0.169
Education Sector	χ ^2^ (6) =6.000, p=0.423	χ ^2^ (16) =21.333, p=0.166	χ ^2^ (15) =15.111, p=0.443
Others	χ ^2^ (35) =64.800, p=0.002 *	χ ^2^ (40) =45.667, p=0.248	χ ^2^ (24) =41.550, p=0.014 *

* Good level of significance (p < 0.05).

### Concerns of primiparous mothers towards newborn health and development

**Figure 5.**
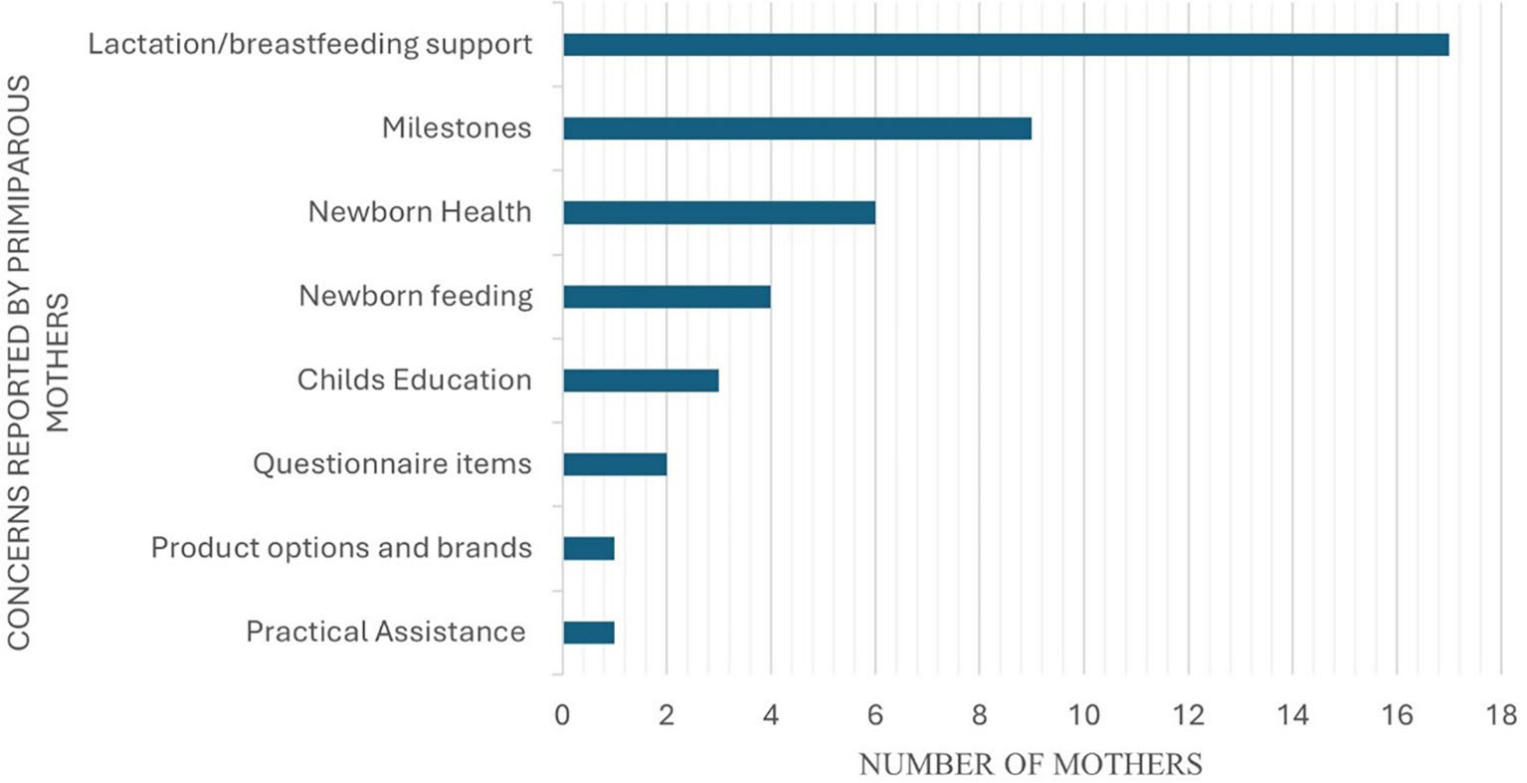
Concerns of primiparous mothers on newborn health and development.

Following the administration of the AQ (pre-test), a variety of responses were obtained from primiparous mothers through open-ended questions pertaining to concerns on newborn care and development as well as their perceived importance of communication with the newborn. Figure 5 represents the identified concerns of primiparous mothers about newborn development.

### Perspectives of primiparous mothers towards the importance of communication skills during infant development

A certain proportion of primiparous mothers (5%) reported that communication with their infants will (1) obtain better responses from the newborn, (2) facilitate in general and intellectual development of the newborn, (3) develop social skills, (4) encourage play behaviours in newborns, and (5) improve mother-child bonding. However, a few mothers (2%) reported that communication with their newborns may not necessarily be important in the early years but becomes crucial only when the child becomes older. Additionally, some mothers (3%) reported to be unaware of the importance of communication with the newborn.

### Feedback of primiparous mothers towards the N-CHFSEP

The results of the FQ revealed 67%, 32% and 1% of primiparous mothers to strongly agree, agree, and neutrally agree respectively towards the effectiveness of the education program. Following the administration of the FQ, a variety of responses were gathered from the open-ended question (Item no. 6). Several mothers (50%) reported that N-CHFSEP benefitted them by (1) providing additional information on communication skills, (2) offering a comprehensive view on the timelines of the developmental process, (3) emphasizing the importance of hearing, (4) addressing feeding concerns, and (5) providing insights into the newborn health. In addition, the programme was effective in (6) establishing the common disbelief that typically male children may verbally communicate much later than female children, and (7) understanding the behaviours to be observed during their newborn development. Others expressed a need for specific details pertaining to (8) fever management, (9) troubleshooting lactation failure and its effect on the baby, (10) details on avoidance of the type of feeds for the infant, and (11) developmental aspects beyond 6 months were reported by 8%, 15%, 5%, and 5% of the mothers respectively.

## Discussion

The result of the current study portrays the confidence levels of primiparous mothers before and after being subjected to the N-CHFSEP, and the possible influencing variables (age, education, family type, and occupation) on the same. The results are discussed below:

### Newborn communication skills

A notable increase in the number of mothers (not all) reporting heightened confidence levels following receiving the N-CHFSEP was observed. This observed change (pre and post) was statistically significant as per paired t-test analysis (p <0.05) (as reported in Table 2). Considering the questions (in AQ) pertaining to communication skills, 89 (for Item 1) and 84 (for Item 2) primiparous mothers reported having a pre-existing understanding (pre-AQ administration) of these skills, attributing this to their exposure to a variety of informational sources (educational programs, books, social media platforms, etc.) which may have helped them prepare well in advance for judging their newborn communication skills. In an Australian study, the researchers reported variability in the knowledge of primiparous mothers on early communication development and found them to often rely on friends and social media for information, which was mostly deemed unreliable according to maternal reports. ^6^ Catering to the perspectives of primiparous mothers towards the importance of communication skills in the current study, it was reported that communication with newborns helps them to obtain responses, facilitate in general and intellectual development of their newborn, develop social skills, encourage play behaviours in newborns, and improve mother-child bonding. However, a few mothers reported that communication with their newborns may not necessarily be important in the early years but becomes crucial only when the child becomes older. Additionally, some mothers reported to be unaware of the importance of communication with the newborn. As per the concerns obtained (AQ-Item 18) from the participants of the current study, certain mothers reported to incorrectly estimate the developmental milestones in newborns which was reported in several other studies. ^28^ Additionally, several mothers reported a lack of communication between healthcare professionals regarding newborn language development. ^2^ However, the evident increase in the confidence levels (p<0.05) of the primiparous mothers in identifying the newborn communication skills in the current study (AQ-Item 1 and 2), can be attributed to the increased awareness these primiparous mothers would have received by the N-CHFSEP. As per the N-CHFSEP, the domains specified pertaining to communication development tapped upon milestones including the presence of a social smile, recognition of the mother, patterns of vocalization and babbling and communicative behaviours. The provision of such information may have imparted new knowledge on their newborn communication behaviours leading to increased confidence levels (p <0.05) as reported in Table 2. A randomized control trial done to study the effectiveness of educating parents on infant language development using a TMV Newborn program, in conjugation with a newborn hearing screening program found parents to have significantly enhanced knowledge about early language environments and caregiver responsiveness in promoting infant cognitive and language development. ^2^

Ninety-two mothers reported a heightened increase in the confidence levels in identifying their newborns hearing abilities (AQ-Item 3) prior to the education program. This could be attributed to their awareness of the existing policies and implementation of newborn hearing screening programs available in Indian hospitals ^29^ (including the one in which the participants of the current study were admitted for delivery), resulting in mothers being aware of the risk factors for hearing loss. Following the N-CHFSEP delivery to the participants of the current study, an increase in their confidence levels (p <0.05) for Item 3 was observed. The N-CHFSEP items pertaining to this aspect (on hearing loss) were directed to the knowledge and understanding of the timeline when their baby would ‘appear to listen and pay attention to sounds’, ‘localization of the speaker’, and ‘recognizing and responding to their name call’. An increase in the early detection of hearing loss with a corresponding improvement in language skills, behaviours, and the quality of life of infants due to universal newborn hearing programs has been reported. ^30^

Considering the mothers confidence levels in independently identifying the warning signs (AQ-Items 5 and 7) during the infant’s communication development, an increase in their levels was observed following the N-CHFSEP delivery. This may be attributed to the aspects of N-CHFSEP which included warning signs on ‘social smile’, ‘types of child response’, ‘attentional skills’, and ‘patterns of vocalizations’ along with the corresponding timelines, thereby helping them in identifying potential issues in newborn development. The program also provided mothers with strategies to be used at home to increase communication skills in newborns, thereby increasing their confidence levels. Previous studies have proclaimed a potential lack of awareness among caregivers on implementing communication strategies. ^31^ There is a scarcity of studies focusing on the danger signs of newborn communication development skills in India. Hence, the provision of N-CHFSEP in the current study enhanced maternal confidence levels in identifying and responding to these skills.

### Newborn feeding-swallowing skills

A notable increase in the number of mothers (not all) reporting heightened confidence levels in feeding-swallowing skills following receiving the N-CHFSEP was observed. This observed change (pre and post) was statistically significant as per paired t-test analysis (p <0.05) (as reported in Table 2). Considering the questions (in AQ) pertaining to feeding-swallowing skills, 89 (for Item 4) and 93 (for Item 12) primiparous mothers reported having a pre-existing understanding (pre-AQ administration) of these skills. This may be attributed to various factors such as (1) their personal experiences in observing feeding newborns within the family circle, (2) mothers prior to delivery may have self-educated themselves about feeding skills from other support networks, and (3) the availability of lactation counsellors in hospitals offering mothers with practical assistance and opportunity to discuss feeding related concerns during the hospital stay. This aligns with previous findings in India, which reported that 40% of the mothers received antenatal education on breastfeeding practices, while 61% were educated by family and friends. ^32^ The study also reported that the majority of the mothers had good knowledge about breastfeeding and the nutritional advantages of the same, but only 45% of the mothers practised the knowledge they gained through these sources. As per the concerns obtained (AQ-Item 18) from the participants of the current study (as indicated in Figure 5), certain mothers reported concerns about breastfeeding knowledge and support. These included concerns towards deciding the feeding position, feeding duration, feeding schedule, feeding precautions, post-feeding management, aspiration management, and the type of feeds. The evident increase in the confidence levels (p <0.05) of the primiparous mothers (in the current study) in identifying newborn feeding schedules and understanding of exclusive breastfeeding for six months (AQ-Item 4 and 12), can be attributed to the adequate information provided in N-CHFSEP such as ‘exclusive breastfeeding’, ‘burping’, ‘feeding duration’ and ‘schedule’, and ‘awareness on the usage of formula feeds’. This provision of information likely complemented their existing knowledge of newborn feeding and swallowing skills. Moreover, practical demonstrations by lactation counsellors further reinforce their understanding, contributing to an increase in their confidence levels as reported in Table 2. This finding is consistent with the findings of a systematic review done on the effectiveness of educational programs related to breastfeeding and supportive programs for primiparous mothers. The studies reported the mothers to have increased breastfeeding knowledge and self-efficacy, thereby increasing the frequency of breastfeeding. ^33^

Considering the questions (in AQ) pertaining to the warning signs (AQ-Items 8 and 9) in feeding-swallowing skills, there was a decrease in the number of mothers (77 and 81) reporting to be confident (before N-CHFSEP) compared to items 4 and 12 (89 and 93). This decrease may be attributed to various factors such as lack of previous experiences in newborn care, fear of misinterpretation of potential warning signals, increased stress levels during the early motherhood period, limited access to antenatal and postnatal services, and information overload during the post-pregnancy period. These factors may lead to a state of confusion in mothers, making it difficult to distinguish and retain essential information related to newborn care feeding skills. This contradicts to a previous study conducted in Punjab which reported that 49.6% of urban mothers have a good knowledge of newborn danger signs. ^34^ Following the N-CHFSEP delivery to the participants of the current study, an increase in their confidence levels (p <0.05) for Items 5 and 7 was observed which could be attributed to the program. The mothers were provided with adequate information on the neonatal danger signs such as ‘struggling to swallow’, ‘feeds coming out of the baby's nose immediately after being fed’, ‘vomiting of a large amount of feeds’, ‘coughing immediately after feeds’, ‘baby appearing dull and unable to take feeds’, and ‘forehead sweating immediately after feeds’. This is in line with a previous study done in South India, which included newborn feeding and health danger signs. ^35^ The study demonstrated the effectiveness of a structured teaching program in improving the knowledge of postnatal mothers on newborn danger signs including those related to feeding and newborn health.

### Newborn general health

A notable increase in the number of mothers (not all) reporting heightened confidence levels in newborn health following receiving the N-CHFSEP was observed. This observed change (pre and post) was statistically significant as per paired t-test analysis (p <0.05) (as reported in Table 2). Considering the questions (in AQ) pertaining to newborn health (AQ-Items 10, 11, 13 and 14), primiparous mothers reported having a pre-existing understanding (pre-AQ administration) of these skills. This may be attributed to the information provided on newborn hygiene and health status by the health professionals during their follow-up visits during the post-pregnancy/natal period. These heightened confidence levels may also be attributed to the significant role played by caregivers demonstrating and providing practical training on newborn care skills and the availability of educational cards such as Mother Child Protection Cards helping mothers to track newborn development, immunization schedules, and its importance. These findings were reaffirmed in another study done in South India, where 99.5 % of first-time mothers were aware of the importance of immunization for their children from various sources. ^36^ Some of the concerns expressed by the participants of the current study (AQ-Item 18), were regarding their child's future education, selection of high-quality products to ensure child safety, precautions towards general health, weight gain, and bathing care. Following the N-CHFSEP delivery to the participants of the current study, an increase in their confidence levels (p <0.05) for Items 10,11, 13 and 14 was pertaining to newborn health observed in the mothers. This could be attributed to the N-CHFSEP which included adequate information on identifying the appropriate vitamin supplements, newborn hygiene practices, and post-immunization effects. Though the mothers were confident and aware of these aspects for their newborns, the N-CHFSEP supplemented their knowledge by providing comprehensive information on the administration timeline and schedules on the same. This aligns with previous findings which indicated the effectiveness of flipchart-assisted maternal education in improving breastfeeding practices, hygiene skills, and temperature maintenance in newborns after the educational intervention. ^37^ Considering the mothers confidence levels in independently identifying developmental milestones and precautions towards hypothermia (AQ-Items 15 and 16), there was an increase in the number of mothers (91 and 92 mothers respectively) exhibiting heightened confidence levels following the N-CHFSEP delivery, compared to AQ-Items 10, 11, 13 and 14. This increase in confidence levels could be attributed to the N-CHFSEP, which provided adequate information offered in the child-rearing practices such as monthly milestones, and suggestions to prevent hypothermia. In contrast to many educational programs that do not emphasize comprehensive monthly milestones, especially those that are not verbally presented to these mothers, the N-CHFSEP in the current study was provided via a one-to-one educational session, which was advocated by previous research as well. ^27^ The findings of the current study were consistent with a previous study done in the USA, where the researchers found a higher score in newborn care knowledge and maternal confidence after providing a Newborn Class program on developmental milestones and medical topics such as infant feeding and nutrition, infant health, and hygiene and infant well-being. ^3^

Apart from the earlier mentioned reasons, the overall increase in the confidence levels of primiparous mothers in the current study could be attributed to the timing of intervention (provided second day of post-delivery), the expertise and the training provided by the health care professionals (SLP and Paediatrician), and the structure in which the education program was provided (monthly milestones, suggestions and danger signs) could have significantly contributed to the mother’s knowledge and confidence on early newborn caring skills, which was reiterated in previous studies. ^37^ The majority of the participants in the current study were recruited from Government Lady Goschen Hospital, Mangalore which is exclusive for maternity care, encompassing a team of health care professionals from various disciplines. Studies have emphasized the significance of healthcare professionals as the primary source of information when queried about the benefits of postpartum educational programs. ^3^ In addition, the hospital (in the current study) offered several instructional resources in Kannada and English such as posters on early newborn care skills and Mother Child Protection cards which may have supplemented their confidence levels.

### Sociodemographic factors

According to the results of the chi-square analysis, age, education, occupation, and family type had a significant effect (p<0.05) on the confidence levels before and after the administration of the N-CHFSEP. The maternal age (18-25 and 26-35 years) presented an effect (p <0.05) on the confidence levels in communication skills, feeding-swallowing skills, and newborn health as reported in Table 3. The higher confidence levels observed among both young and older mothers could be attributed to their inclination to use social media and mobile applications, ^38^ which are generally free and readily available sources of information about newborn care. Such applications enable mothers to get information on monthly milestones, paediatric weight gain, vaccination charts, reminders on health checkup days, etc. Access to such information could contribute to increased confidence levels in managing various domains of communication skills, feeding-swallowing skills, and newborn health. Research on the utilization and effects of the Baby Buddy App, among first-time mothers revealed that the application provided easily accessible and reliable pregnancy information aiding them in effectively communicating with healthcare professionals. ^39^

Maternal education levels presented a significant effect (p <0.05) on the confidence levels before and after the administration of the N-CHFSEP as reported in Table 3. The higher educational levels (Degree, <=10 ^th^ Std, <=12 ^th^ Std) were observed to have an effect (p <0.05) on the confidence levels of newborn communication skills and general health whereas the lower education level (<=10 ^th^ standard) specifically presented a significant effect (p <0.05) on the confidence levels in feeding-swallowing skills. This could be attributed to a deeper understanding of newborn care among highly educated mothers because of their formal education and exposure to various informational resources. A study on maternal educational levels reported to be significantly associated with the knowledge, attitude, and practice in newborn care. ^40^ They found mothers with no formal education to score less than those who had higher education, which was corroborated by others as well. Higher levels of education may cause an urge among these mothers to obtain evidence-based resources from reliable websites and scientific journals. This inclination may prompt them to seek clarification from qualified healthcare professionals when needed. For example, considering communication skills, mothers may look for strategies such as purchasing specialized toys to enhance newborn communication abilities. They may also critically evaluate the information for its validity and relevance before implementing it. Higher education levels may help them make decisions on newborn health resulting in higher confidence levels among these mothers.

According to the results reported in Table 3, a significant effect (p <0.05) on maternal confidence levels on overall newborn care and development was observed among those who belonged to a joint family type. With a joint family structure generally comprising grandparents (experienced caregivers) or extended families, it becomes natural for first-time mothers to absorb traditional methods of newborn care along with practical demonstrations, thereby boosting their confidence levels. Regular interaction and discussions by these caregivers provide mothers with practical suggestions and solutions in various aspects of newborn care. The joint family type facilitates obtaining collective information on effective parenting strategies and decision-making during certain newborn practices such as feeding, hygiene practices, and newborn health practices, reassuring the first-time mothers in the process.

Primiparous mothers from nuclear families presented a significant effect (p <0.05) on confidence levels concerning general newborn health as reported in Table 3. This might be because of the greater responsibility mothers in nuclear families may experience towards newborn care, without the presence of the extended family members. To overcome such challenges, mothers trained themselves to become competent in newborn care practices. The provision of N-CHFSEP in the current study may have addressed these maternal concerns by equipping them with monthly milestones, newborn care suggestions, and monthly warning signs, thereby causing an effect on their confidence levels. Hence it is important to consider the family structure when developing interventions aimed at enhancing maternal confidence in newborn care.

Maternal occupations such as homemakers and others (government and private officials) were found to have a significant effect (p<0.05) on the confidence levels concerning newborn communication skills, feeding-swallowing skills and newborn health as reported in Table 3. Homemakers generally get opportunities to engage in interactions with their newborns thereby fostering newborn language development and communication skills. Their consistent presence at home provides opportunities for monitoring newborn care and interventions when required causing increased confidence levels among mothers pertaining to these skills. As reported in Table 3, only homemakers when compared to other occupations showed a significant effect (p<0.05) on newborn feeding-swallowing skills. Although being a homemaker is highly advantageous, there do exist challenges such as feeling isolated, lacking professional advice, and feeling financial constraints, making them less confident in newborn practice in the process. Primiparous mothers with government and private jobs may face unique challenges in balancing work with their caregiving duties. Access to resources such as government-sponsored healthcare programs or childcare services, could positively impact their ability to prioritize feeding skills development. The provision of N-CHFSEP, which involved comprehensive information on newborn caregiving skills might have complemented and enhanced the caregiving practices of both homemakers and government officials. In contrary studies have found no significant association between mothers' occupation and newborn care. ^40^^,^^41^

### Feedback on N-CHFSEP delivery

It becomes important to obtain feedback (FQ Item 6) from primiparous mothers for a comprehensive understanding of the strengths and weaknesses of the developed N-CHFSEP. As per the feedback obtained (FQ- Item 6) from the participants of the current study, the N-CHFSEP provided mothers with additional information in various aspects regarding communication skills, emphasized the importance of hearing, and corrected misconceptions. Studies have found that feedback of educational programs has positive outcomes such as encouraging mothers to interact and read with their infants in a responsive manner. These mothers reported being willing to recommend the education program to others and apply the knowledge with their newborns at home (2). As per the feedback obtained (FQ- Item 6) from the participants of the current study, concerning newborn general health, the N-CHFSEP provided insights into newborn health, offering a comprehensive view of developmental timelines, and understanding the behaviours to be observed during their newborn development. A study on the NewBorn Class program reported that it benefitted approximately 58% of the mothers by providing comprehensive knowledge for first-time mothers, and providing information on medical topics such as feeding, diaper rash, and developmental skills (3). The mothers of the current study also demanded for additional information on fever management, troubleshooting lactation failure and its effect on the baby, details on avoidance of the type of feeds for the infant, and developmental aspects beyond 6 months. Furthermore, some mothers highlighted the need for the practical application of the information provided. They also suggested supplementing the N-CHFSEP through practical demonstrations to enhance their confidence levels in providing neonatal care. This feedback emphasizes the importance of practical learning experiences, suggesting hands-on demonstrations to be useful additions to future educational programs.

Although the developed N-CHFSEP showed a significant positive change (pre and post-administration) in the attitude levels of the mothers, having a control group (mothers without receiving the intervention program) and comparing them to an experimental group (mothers receiving the intervention program) would have increased the validity of the tool. Additionally, the effectiveness of N-CHFSEP was determined immediately after the administration, leaving uncertainty about information retention up to 6 months post-intervention, which could be controlled for in future studies. Moreover, the study did not assess whether participants practically applied the knowledge gained from the N-CHFSEP in their daily caregiving routines, potentially limiting the effectiveness of the intervention. The N-CHFSEP included information on neonatal care from only two disciplines (SLP and Paediatrics), suggesting the possible inclusion of additional members (such as fathers, grandparents, lactation counsellors, psychologists, ASHA workers, nurses, etc.) in future studies to provide a more comprehensive understanding of various aspects of newborn health and development. The effectiveness of the N-CHFSEP could be enhanced by incorporating practical demonstrations or hands-on training sessions during administration which were among the demands by the primiparous mothers of the current study.

With the implementation of the N-CHFSEP in the current study, the primiparous mothers received a comprehensive education on various aspects of newborn skill development. The observed increase in confidence levels following N-CHFSEP suggested the program to effectively complement the mother's knowledge and understanding, thereby contributing to improved confidence and awareness regarding newborn care. This understanding is crucial for the implementation of comprehensive educational interventions addressing the specific needs of primiparous mothers as it may lead to enhanced maternal and infant health outcomes.

## Ethics and consent

The study was approved by the Institutional Ethics Committee (IEC KMC MLR 03/2023/108) of Kasturba Medical College, Mangalore, Manipal Academy of Higher Education on 20.07.2023 and was registered under the Clinical Trials Registry of India (CTRI/2023/05/053109) on 25.05.2023. The researcher obtained written informed consent from the participants followed by their socio-demographic details.

## Data Availability

*Name of the repository*: Mendeley Data *Project title*: Estimating the efficacy of Newborn-Communication, Health, Feeding and Swallowing Education Program (N-CHFSEP) for primiparous mothers. ^42^ https://data.mendeley.com/datasets/7c75ws94vn/2 This project contains the following underlying data:
• DATA ENTRY SHEET.xlsx (includes data on the patient demographics, responses from pretest Attitude Questionnaire, posttest Attitude Questionnaire, and Feedback Questionnaire) • Read Me.txt (description to understand the variables in data files) •
completed_CONSORT_checklist.docx (includes the CONSORT checklist)
*License*: Creative Commons Attribution 4.0 International license (CC-BY 4.0).
