# Evaluating the Efficacy of the Newborn Communication, Health, Feeding, and Swallowing Education Program (N-CHFSEP) for First-Time Mothers in Mangalore taluk, Dakshina Kannada district, Karnataka, India: A Preliminary Study

**DOI:** 10.12688/f1000research.152320.2

**Published:** 2025-01-31

**Authors:** Deepthi Ouseph, Jayashree Kanthila, Sunil Baliga, Shraddha Shetty, Sudhin Karuppali

**Affiliations:** 1 Department of Audiology and Speech Language Pathology, Kasturba Medical College Mangalore, Manipal Academy of Higher Education, Karnataka, Manipal, 576104, India; 2 Department of Pediatrics, Kasturba Medical College Mangalore, Manipal Academy of Higher Education, Karnataka, Manipal, 576104, India; 3 Department of Pediatrics, Yenepoya Medical College, Yenepoya University, Mangalore, Karnataka, 575018, India; 4 Department of Obstetrics and Gynecology, Kasturba Medical College Mangalore, Manipal Academy of Higher Education, Karnataka, Manipal, 576104, India; 5 School of Rehabilitation and Medical Sciences, College of Health Sciences, University of Nizwa, Nizwa, Oman

**Keywords:** accessible education, child health, early childhood development, health education, mental health, primiparous mothers

## Abstract

**Background:**

Primiparous mothers face diverse challenges during pregnancy and post-childbirth. There is limited data on the effectiveness of postnatal educational programs for new mothers (Subramanian et al., 2020). This study aimed to assess the efficacy of an educational program designed to improve primiparous mothers’ attitudes toward newborn communication, general health, feeding, and swallowing. The objectives were (1) to develop an attitude questionnaire (AQ), a parent education program [Newborn Communication, Health, Feeding and Swallowing Education Program (N-CHFSEP)], and a feedback questionnaire (FQ); and (2) to estimate the efficacy of N-CHFSEP pre- and post-delivery. The research, conducted between August 2023 and February 2024, involved 98 primiparous mothers who gave birth to healthy newborns through any delivery method. Participants were proficient in English or Kannada. The study excluded mothers delivering multiples, those with significant medical or psychological issues, and health professionals.

**Methods:**

The study had two phases: Phase 1 developed and validated the AQ, N-CHFSEP, and FQ. N-CHFSEP covered key topics, including milestones in communication, feeding, and general health from birth to 6 months. Phase 2 administered these tools to participants. The data was analyzed using descriptive statistics, paired t-tests, and chi-square tests.

**Results:**

93% of mothers reported increased confidence in recognizing communication, feeding, and health-related signs in their infants after completing the program. This change was statistically significant (p < 0.05). Sociodemographic factors like maternal age, education, occupation, and family type significantly influenced confidence levels. Feedback from participants highlighted the program’s effectiveness in enhancing knowledge, with suggestions for improvements.

**Conclusions:**

N-CHFSEP significantly boosted mothers’ confidence in newborn care, contributing to better maternal and infant health. However, the study lacked a control group, and the effectiveness was evaluated only immediately after the program. Future research could incorporate practical demonstrations and hands-on training to further enhance the program’s impact.

## Introduction

Pregnancy and childbirth represent significant milestones in a woman’s life, permanently altering her identity and way of living in a continuous and dynamic manner ( Kuipers et al., 2021). Primiparous mothers (first-time mothers) face a wide range of emotions including joy, excitement, and anxiety, alongside overwhelming and stressful experiences such as routine newborn care, breastfeeding difficulties, lack of sleep, and physically taxing household duties ( Nan et al., 2020). The postpartum recovery process involves recuperating from the physical and emotional damage and acclimating to the demands of caring for a newborn, which can be challenging for many first-time mothers. Feelings of inadequacy and self-doubt can exacerbate especially for new mothers who feel unprepared for the demands of parenthood or compare themselves to others ( Saharoy et al., 2023). The fact that many first-time mothers have never held a newborn before can be intimidating. The neonatal period becomes an important stage for brain development demanding a significant level of care from caregivers, facilitating the formation of new connections within the brain ( Winston & Chicot, 2016). The child’s growth and development is guided by the attainment of various developmental milestones (cognitive, gross, and fine motor, language, and social-emotional and behavioral). The ability of caregivers to effectively identify these milestones facilitates early interventions, improving overall health outcomes ( Scharf et al., 2016). Concern over whether they are doing these duties right can result from the overwhelming nature of caring for a newborn. Fearful of making mistakes, new mothers often doubt their skills and intuition. The mothers’ inexperience makes them feel vulnerable and more prone to thoughts of failure. Recognizing these difficulties is essential as it draws attention to the necessity of all-encompassing support networks that enable mothers to flourish.

In the first year following childbirth, maternal mental health has a significant role in both postpartum and baby health. The maternal confidence, knowledge, and attitudes play an important role during this period ( Suskind et al., 2018). A mother’s belief that she can comprehend and meet her child’s needs and possess the knowledge and skills necessary to provide for them encompasses maternal confidence ( Arante et al., 2020; Badr, 2005). A good transition to motherhood during parental role adjustment is indicated by this confidence level ( Arante et al., 2020; Goto et al., 2010). Reduced levels of confidence in primiparous compared to multiparous mothers negatively impact their ability to provide infant care ( Gozali et al., 2020). A mother’s self-confidence is influenced by a variety of elements, including contextual traits like social support, the temperament of the child, and the mother’s mental health ( Leerkes & Burney, 2007; Oswalt & Biasini, 2012). Childhood interactions with caregivers, particularly mothers, have an impact on a child’s social, emotional, and cognitive development ( Kuo et al., 2009). Maternal health literacy programs influences a woman’s motivation and capacity to seek, comprehend, and apply information to enhance the well-being of both mother and child ( Nutbeam et al., 2018). Such programs provide newborn health indicators, especially useful for primiparous mothers as they encounter feelings of frustration and uncertainty, struggling to identify specific reasons for their infant’s issues. Consequently, most new postpartum mothers express a strong desire for professional guidance and support in both infant and self-care ( Nan et al., 2020). Studies on the needs of primiparous mothers have indicated their efforts to fulfil their informational requirements regarding pregnancy, through attending educational sessions and utilizing online resources to gather the necessary information. Numerous studies on primiparous mothers have highlighted the importance of educational programs delivered by healthcare professionals assisting in newborn care and the implementation of proper procedures to promote infant health ( Shorey et al., 2018; Turk Dudukcu & Tas Arslan, 2022).

Programs such as the Maternal and Newborn Care Intervention (MNCI) program on maternal functioning revealed its efficacy in improving primiparous mothers functioning during the postpartum phase ( Thwin et al., 2023). Similarly, Gozali and colleagues evaluated the impact of a Newborn Education and Discharge Class, and found mothers who took the programme to significantly exhibit higher levels of knowledge than the control group ( Gozali et al., 2020). The “HUG Your Baby” parent education and support program, indicated the parent education and support program to foster the parent’s general well-being, offering them the needed tools to ensure a seamless transition from the NICU to home, thereby providing them with confidence in their position as carers ( Hunter et al., 2019). Several other universally used maternal literacy programmes have been reported to be used (1) the “Learn the Signs. Act Early.” (LTSAE) programme of the U.S. Department of Health and Human Services and the Centers for Disease Control and Prevention, (2) the Mount Sinai Parenting Center ( Gozali et al., 2020), (3) the Internet Newborn-Care Education Programme (INCEP) developed in Taiwan ( Kuo et al., 2009), (4) the postnatal psychoeducation programme developed in Singapore ( Shorey et al., 2015), and (5) an educational support program developed in Iran ( Kermanshahi, 2017). The Early Childhood Development (ECD), a UNICEF global programme in India included schemes such as (1) the Home-Based Care for the Young Child (HBYC) developed by the National Health Mission as an expansion of the Home-Based Newborn Care (HBNC) programme, (2) the HBNC+ (an extension of HBNC) ( Newton-Lewis & Bahety, 2021), (3) the Rashtriya Bal Swasthya Karyakram (RBSK), which is an initiative of the Government of India’s Ministry of Health and Family Welfare, and (4) an updated version of the Mother and Child Protection (MCP) Card ( Mahyavanshi et al., 2022).

Although numerous educational programs exist, there is a lack of a holistic approach that comprehensively addresses newborn communication, feeding, swallowing, and general health. The awareness of the development of early communication skills among primiparous mothers suggests a need for more reliable and approachable information about the development of communication skills ( Williams et al., 2014). Feeding and swallowing skills are considered promoters of physical, mental, and psychological health, providing infants with adequate nutrition required for development, which when unmet, may cause changes in the brain structure leading to general health challenges, cognitive deficits, and other metabolic, and behavioral difficulties. Monitoring these developmental milestones assumes a critical role in recognizing deviations and facilitating prompt interventions ( Scharf et al., 2016). This emphasizes the need for a comprehensive educational module addressing a range of important developmental aspects for mothers with their newborns. Access to such reliable sources of information empowers primiparous women to enhance their confidence and shape their mental expectations and ideals regarding pregnancy and childbirth. Additionally, such programs would enable them to feel more assured and better formulate mental expectations and principles concerning their pregnancy and childbirth experiences ( Fenwick et al., 2015). There are only a handful of centres that mandatorily provides such information to primiparous mothers with a dearth of existing studies in India pertaining to the efficacy of using such education programs.

Despite the availability of various educational initiatives, there is a noticeable gap in comprehensive programs that address all critical areas of newborn development. This study aims to develop and validate a comprehensive educational program and assess its efficacy in enhancing maternal confidence and knowledge among primiparous mothers. The current study aimed to explore the efficacy of an educational program on the attitude of primiparous mothers towards milestones of early infant communication, feeding-swallowing and general health of their infants. The objectives of the study were (1) to develop and validate an attitude questionnaire, parent education program and feedback questionnaire; (2) and to estimate the efficacy of an education program pre and post-intervention delivery.

## Methods

The research was a before-after comparison study carried out between August 2023 and February 2024. A non-random convenience sampling method was adopted for the study. The study was approved by the Institutional Ethics Committee (IEC KMC MLR 03/2023/108) of Kasturba Medical College, Mangalore, Manipal Academy of Higher Education and was registered under the Clinical Trials Registry of India (CTRI/2023/05/053109). By confirming that all necessary elements of the trial design, conduct, analysis, and reporting have been fully addressed, we attest that this study complies with the CONSORT (Consolidated Standards of Reporting Trials) checklist. This dedication encourages our study findings to be transparent, rigorous, and reproducible. We certify that this study was carried out in compliance with the Declaration of Helsinki, guaranteeing that the moral standards for human subjects’ medical research were respected. This commitment entails getting informed consent, protecting participant privacy, and putting the welfare of all study participants first.

### Participants

The sample size was calculated using the formula: $n=\frac{Z_{1-\frac{\alpha }{2}}^{2}\times \mathrm{pq}}{d^{2}}$ ; where Z = 1.96, standard normal at 5% level of significance; p = 0.47%, q= 1-p, d=0.1 ( Varghese et al., 2020). The study followed a single-arm pre-post study design similar to other educational interventional studies ( Maharjan et al., 2022; Matovelo et al., 2022). A total of 98 primiparous mothers [mean age (in years): 26.03 ±3.79] were recruited for the study.

Seventy-six (77.6%) of them belonged to a joint family, while 22 (22.4%) were from a nuclear family. These mothers comprised homemakers (62%), homemakers after having resigned from their jobs due to pregnancy (11%), working in educational sectors (8%), and other private/government sectors (19%). The educational qualifications indicated mothers have completed their basic formal education (38%), higher secondary education (19%), diploma (35%), graduation (35%), and postgraduation (3%).

The inclusion criteria included (1) inpatient primiparous mothers without any prior obstetric history, (2) mothers having given birth to healthy newborns through various delivery methods [vaginal, assisted vaginal (including vacuum, forceps, or cesarean)], and (3) mothers proficient in English or Kannada (a South Indian language spoken in the state of Karnataka). The exclusion criteria included (1) primiparous mothers delivering twins/triplets with significant history of obstetric, medical, or psychological problems, and (2) mothers who were health professionals. Multiparous mothers were excluded due to their experience in caring for newborns. Mothers with obstetric complications were excluded as that may affect their ability to participate in the educational program or effectively express their attitudes. All participants were recruited from Government Lady Goschen Hospital (a government hospital exclusive for maternity care), and Kasturba Medical College Hospital (a multidisciplinary speciality centre) of the Mangalore taluk, Dakshina Kannada district.

### Procedure

The present study was conducted in 2 phases. Phase 1 included the development of an (a) attitude questionnaire, (b) parent education program, and (3) feedback questionnaire; while Phase 2 included the administration of the questionnaires and the education program on the participants, followed by data analysis of the retrieved data.

Phase 1: Development of the tools.

a) Attitude questionnaire (AQ): The AQ was developed by reviewing relevant literature ( Bawazir et al., 2013; Karuppannan et al., 2020; Kujur et al., 2022; Shorey et al., 2018; Varghese et al., 2020) on primiparous mothers' attitudes towards infant communication, feeding-swallowing practices, and general health. The questionnaire was designed to encompass various domains (communication milestones, feeding schedules, warning signs during feeding-swallowing and milestones, newborn hygiene, bathing care, vitamin supplements, immunizations, developmental milestones, and general care). The final version of the questionnaire (Appendix-I) comprised 16 closed-ended statements, each designed to assess maternal confidence in the above-mentioned domains. Items 1, 2, 3, 5, 7 were pertaining to communication skills and represented as C1, C2, C3, C5, and C7 respectively. Items 4, 6, 8, 9 and 12 were related to feeding-swallowing skills indicated as F4, F6, F8, F9 and F12 respectively. Finally, items 10, 11, 13, 14, 15 and 16 focused on newborn health and were indicated as H10, H11, H13, H14, H15 and H16 respectively. Each item was to be rated using a 5-point Likert rating scale (ranging from 5 being extremely confident to 1 being extremely unconfident). Additionally, the questionnaire included three open-ended questions indicated as G17, G18 and G19 targeting the mother's attitudes, concerns and opinions on the importance of communication and the well-being of newborns.
b) Parent Education Program: The parent education program [Newborn-Communication, Health, Feeding and Swallowing Education Program (N-CHFSEP)] was developed after reviewing the existing literature on the developmental milestones of newborns. This provided an initial framework for the education program which was further developed with discussions with experienced pediatricians and speech-language pathologists (SLPs). The program covered essential information incorporating a comprehensive set of monthly milestones (0-6 months) crucial for development. The N-CHFSEP featured a total of 120 statements, each appropriately categorized within the corresponding age range. The following table ( Table 1) depicts the general framework of the N-CHFSEP used in the current study.
c) Feedback questionnaire (FQ): The FQ was developed after reviewing the literature ( Çinar & Öztürk, 2014; Khresheh et al., 2011; Kuo et al., 2009; Meedya et al., 2017) on first-time mother’s feedback towards parent education on infant communication, feeding-swallowing and general health of infants. A conceptual framework was developed which was expanded based on discussions with experienced pediatricians and SLPs. This questionnaire (Appendix-II) contained five closed-ended statements following a 5-point Likert rating scale (ranging from 5 being strongly agree to 1 being strongly disagree). Additionally, there was one open-ended question, allowing mothers to share their suggestions regarding the N-CHFSEP.

**Table 1.** General framework of the N-CHFSEP developed for the study.

Age range (months)	0-1	1-2	2-3	3-4	4-5	5-6
Statements	20	19	19	21	16	20
Age wise suggestions	3	1	0	1	0	0
Total items	23	20	19	22	16	20
General suggestions	12					
Red flags	21					

Content Validation

The AQ, FQ, and N-CHFSEP were subjected to content validation by an expert panel to evaluate their adherence to established scientific facts and the grammatical accuracy of the designed statements. The panel included three primiparous mothers who were professionally qualified speech-language pathologists (SLPs) and three primiparous mothers who were professionally qualified paediatricians, each of them having a minimum of five years of clinical experience. All experts received a copy of the developed questionnaires (AQ and FQ) and N-CHFSEP. Each expert reviewed the tools (item-wise) on a 3-point Likert rating scale (0 being inappropriate, 1 being modifications required, and 2 being appropriate). For the N-CHFSEP validation, the panel evaluated the suitability of the milestones to appear under the corresponding age (0-6 months). Following the content validation by SLPs and the pediatricians, the content validation index (CVI) was calculated at both the item and scale level for the AQ, N-CHFSEP, and FQ respectively. The Item level CVI (I-CVI) was computed by dividing the number of experts who rated the items as one or two by the total number of experts. Scale level CVI (S-CVI) was then calculated by averaging the I-CVI scores for AQ, and FQ. The resulting S-CVI scores were 0.95 for AQ, 0.90 for N-CHFSEP, and 1.0 for FQ. The I-CVI scores for the N-CHFSEP fell below the desired level (0.78), indicating insufficient agreement among experts and, hence demanding a revalidation. The same expert panel participated in this process to obtain an I-CVI score of 1.

The AQ, FQ and N-CHFSEP underwent grammatical modifications (e.g. ‘The child … ….’ to ‘My baby … ….’) to enhance their comprehensibility for mothers. Based on expert advice, the N-CHFSEP was revised to highlight potential warning signs using the checklist released by the Centers for Disease Control and Prevention (CDC) within each month and across the 6 months. Furthermore, the program was revised to incorporate recommendations for fostering the child's communication abilities and holistic growth. Specific suggestions were made to include illustrations of certain elements in the N-CHFSEP, to facilitate better comprehension of the presented content. The SLPs provided valuable input, including guidance on breastfeeding practices, as well as identifying red flags related to communication and feeding milestones. The pediatric experts suggested the reorganization of milestones by months and repeating specific items in their respective months. Additionally, it was recommended to develop a pamphlet on the N-CHFSEP which would serve as a carry-home resource for mothers. Following the incorporations of the modifications the final N-CHFSEP was ready for administration.

Translation

The AQ, N-CHFSEP, and FQ were translated into Kannada (one of the common regional languages spoken in the Mangalore taluk). The AQ, N-CHFSEP, and FQ underwent both forward and backward translations which were performed by a bilingual translator proficient in both Kannada and English, with expertise in the subject matter. Following the completion of both forward and backward translations, a final Kannada version of the AQ, N-CHFSEP, and FQ was ready for administration. A pilot study was conducted on two Kannada-speaking participants to assess the comprehensibility, clarity, and cultural relevance of the finalized translated AQ, N-CHFSEP, and FQ. This continuous process of forward and backward translation contributed significantly to the questionnaire's accuracy and cultural appropriateness.

Phase 2: Administration of the tools and data analysis

The data collection took place in the postnatal ward through one-on-one interactions. Participants were recruited by reviewing their medical records based on the inclusion and exclusion criteria. The data was collected from each of the participants on the second day following their delivery due to the discomfort commonly experienced by the mothers on the first-day post-delivery. Safety and hygiene were strictly adhered to by following hand hygiene protocols and by wearing surgical masks (as per WHO guidelines) before approaching the participants at their bedside. Efforts were made to facilitate participants to be seated in a comfortable position, enhancing effective interaction. The researcher obtained written informed consent from the participants followed by their socio-demographic details. Each participant was oriented to the study goals and objectives and was provided with comprehensive information pertaining to the questionnaires and the education program they will be subjected to. They were actively encouraged to seek clarifications on any uncertainties pertaining to the items within the questionnaire. The study commenced by providing each participant with the AQ which required them to read the questions and mark their level of confidence across each item, followed by the presentation of the N-CHFSEP, during which the examiner verbally presented the participants with the program, further to which a printed version of the N-CHFSEP was provided, after which the AQ was re-administered. Subsequently, feedback regarding the N-CHFSEP was obtained from the participants using the FQ. All verbal responses from the mothers were recorded using a digital voice recorder (Sony ICD-UX560F Stereo IC recorder 4 GB). The total administration spanned a duration of 25-30 minutes.

The digital voice recordings of the primiparous mothers' responses towards the AQ and FQ administration were transcribed and subjected to quantitative analysis. All open-ended questions were subjected to qualitative analysis by categorizing the verbatim into comprehensive themes to examine the responses received. Descriptive statistics (quantitative data) were performed using SPSS version 25.0. The continuous variables were analyzed using mean and standard deviation while the discrete variables were analyzed using frequency and percentage. The efficacy of the N-CHFSEP was estimated by comparing the pre and post-AQ scores and analyzing the FQ responses. A paired sample t-test was performed to determine the confidence levels (before and after intervention) of primiparous mothers across domains of communication skills, feeding-swallowing skills and general newborn health. Chi-square tests were carried out to determine the relationship between the demographics of the primiparous mothers and their confidence levels under each domain (communication skills, feeding -swallowing skills and general newborn health).

## Results

**Figure 1.**
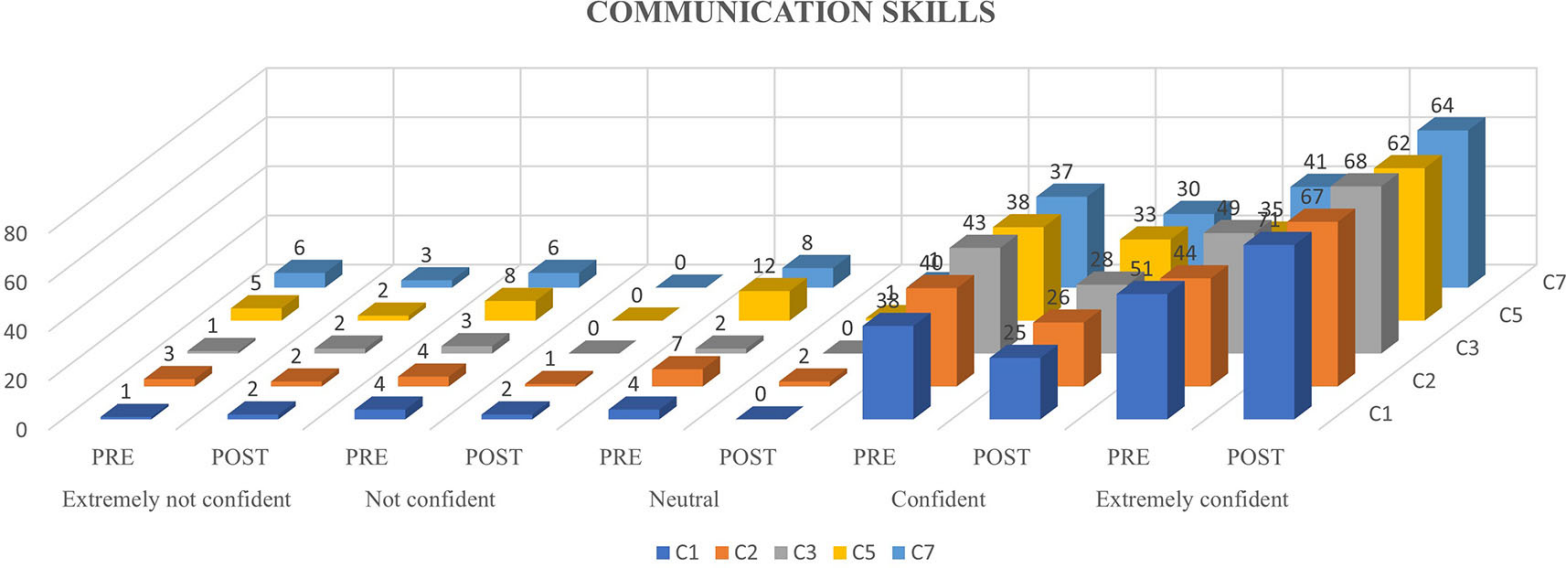
The confidence levels (pre and post administration of the PEP) of primiparous mothers in estimating the communication skills of the newborn.

**Figure 2.**
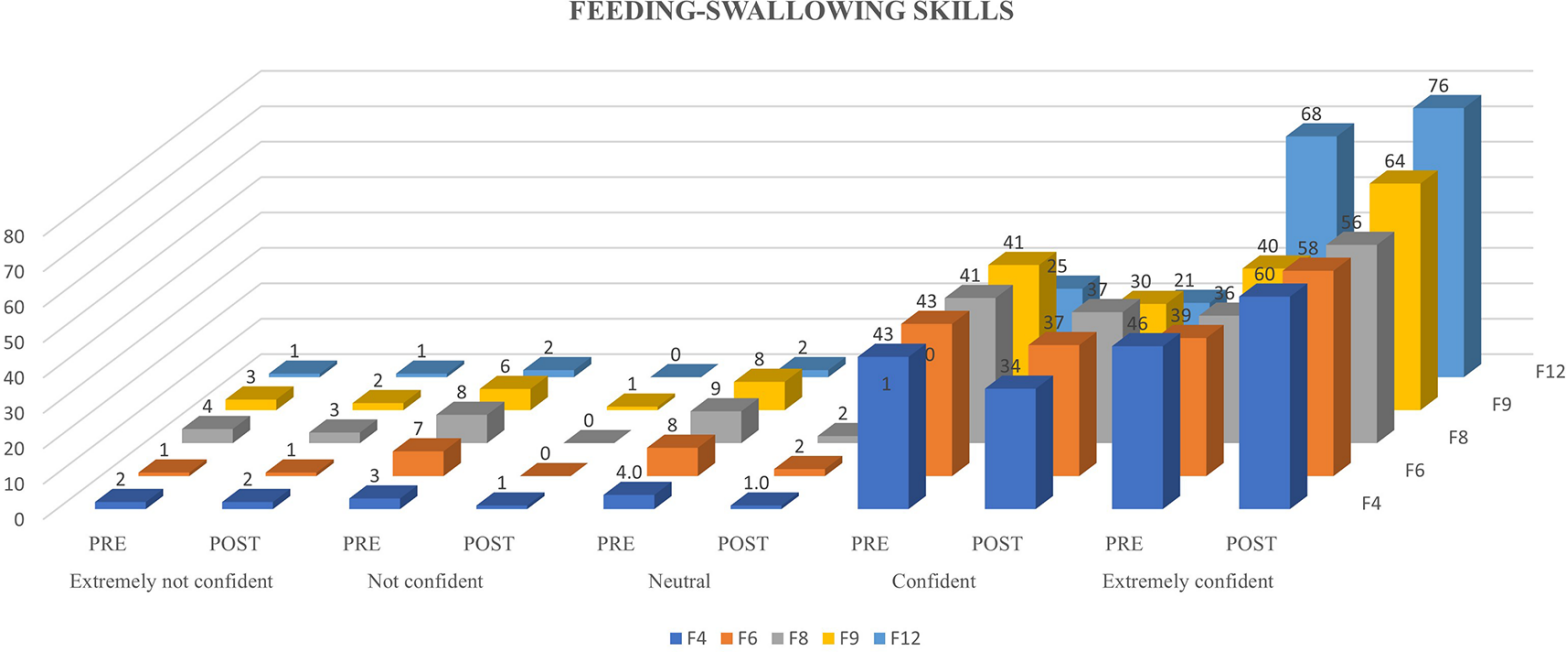
The confidence levels (pre and post administration of the PEP) of primiparous mothers in estimating the feeding-swallowing skills of the newborn.

**Figure 3.**
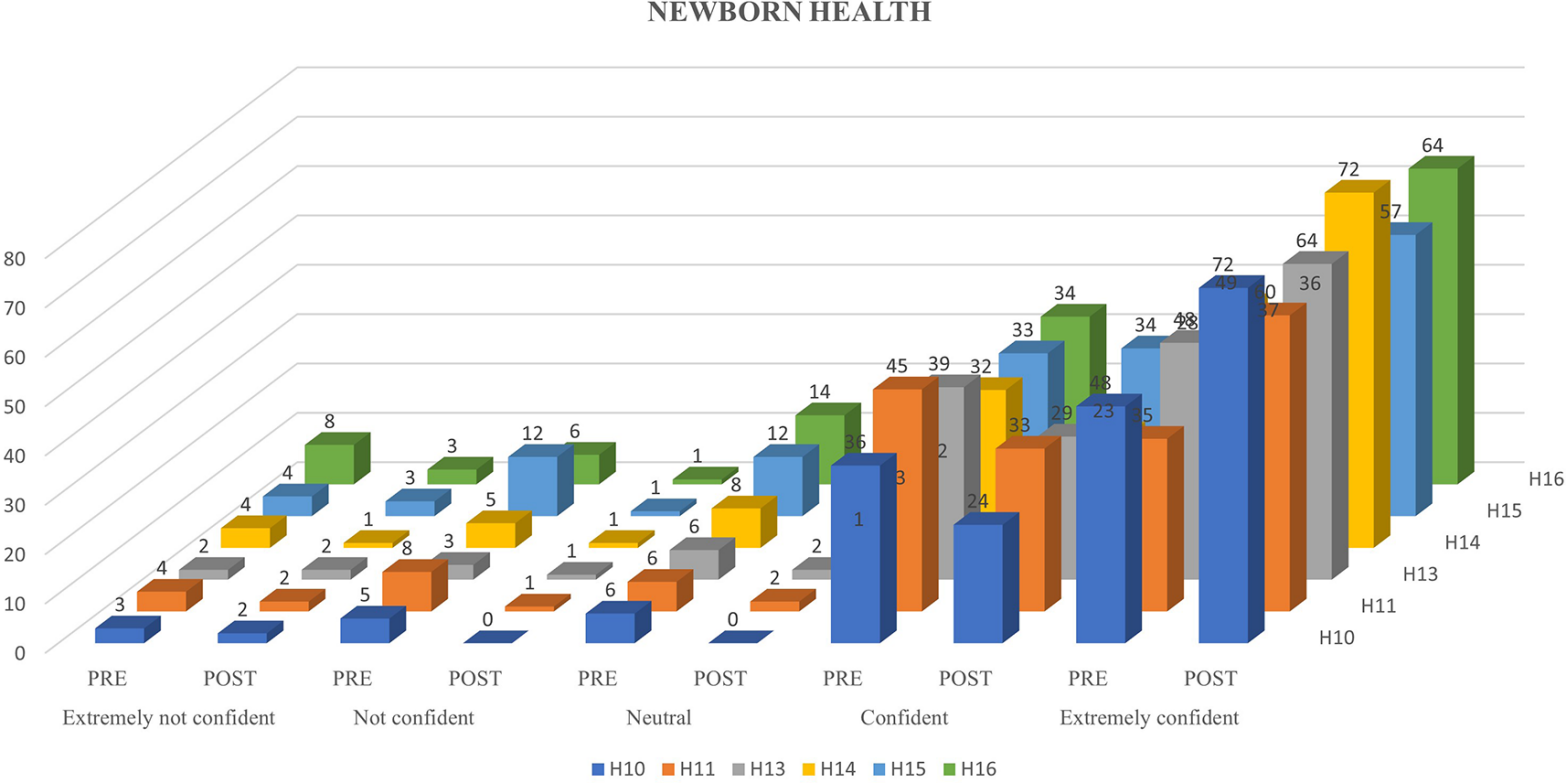
The confidence levels (pre and post administration of the PEP) of primiparous mothers in estimating the health concerns of the newborn.

Ninety-eight participants were involved in the study and underwent the N-CHFSEP. Figures 1, 2 and 3 represents the confidence levels (before and after administration of PEP) of primiparous mothers in judging the communication skills, feeding-swallowing skills, and general health concerns of their infants respectively.

**Figure 4.**
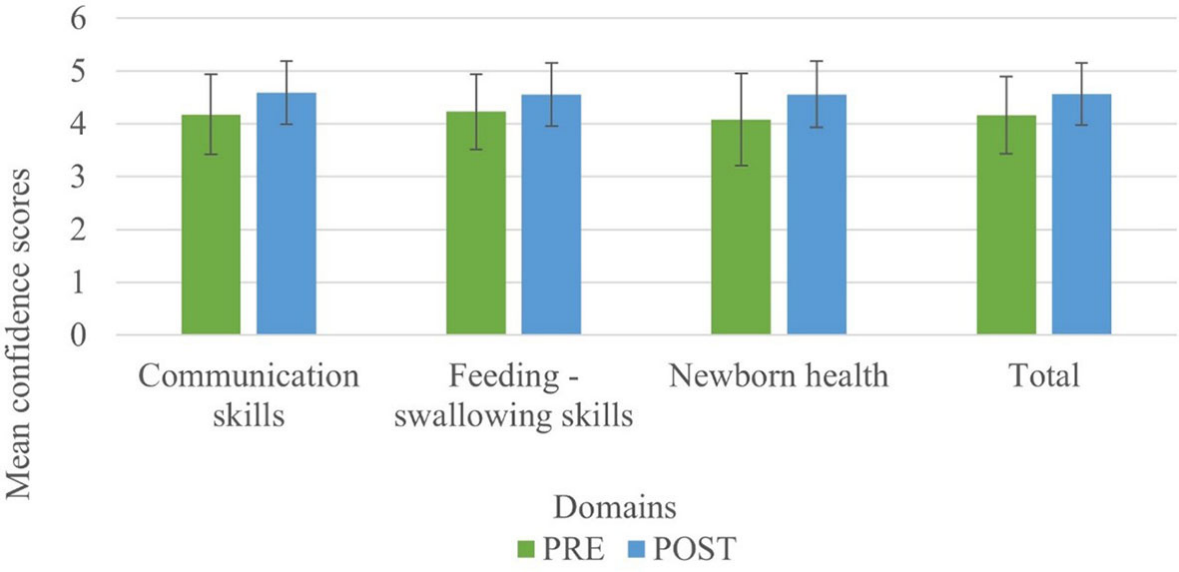
The mean (SD) confidence levels of primiparous mothers before and after the administration of PEP.

Figure 4 represents the before-after confidence levels of primiparous mothers in judging communication, feeding-swallowing, and health concerns of infants.

### Comparison of confidence levels of primiparous mothers following administration of the N-CHFSEP

Table 2 represents the results of a paired sample t-test conducted to compare the effect of N-CHFSEP on confidence levels of primiparous mothers.

**Table 2.** Comparison of total scores of pretest and post-test confidence levels.

Sl. No	Domain	Mean Differences	Std. Deviation	t	Sig. (2-tailed)
1	Communication skills	0.41633	0.69084	5.966	0.000
2	Feeding- swallowing skills	0.33469	0.63361	5.229	0.000
3	Newborn health	0.47959	0.70437	6.740	0.000
	Total Pre-Post	0.41020	0.61271	6.628	0.000

### Effect of age, education, family type, and occupation on the confidence levels reported by primiparous mothers before and after the administration of the N-CHFSEP

The chi-square correlation values between age, education, family type, and occupation towards the total pre-post-test measures of communication skills, feeding-swallowing skills and general infant health are represented in Table 3.

**Table 3.** The pre-post-test chi-square correlation values between participant demographics, and communication, feeding-swallowing, and general health.

Demographics	Chi-square correlation value		
	Communication skills	Feeding – swallowing skills	General infant health
Age			
18-25	χ ^2^ (77) =103.345, p=0.024 *	χ ^2^ (66) =106.669, p=0.001 *	χ ^2^ (105) =177.160, p=0.000 *
26-35	χ ^2^ (104) =240.433, p=0.000 *	χ ^2^ (96) =155.498, p=0.000 *	χ ^2^ (192) =383.548, p=0.000 *
Education			
Degree	χ ^2^ (77) =157.691, p=0.000 *	χ ^2^ (54) =69.190, p=0.080	χ ^2^ (99) =148.575, p=0.001 *
Post degree	χ ^2^ (2) =3.000, p=0.223	χ ^2^ (2) =3.00, p=0.223	---
<=10th STD	χ ^2^ (54) =89.901, p=0.002 *	χ ^2^ (50) =79.001, p=0.006 *	χ ^2^ (91) =158.504, p=0.000 *
<=12th STD	χ ^2^ (40) =61.394, p=0.016 *	χ ^2^ (42) =52.883, p=0.121	χ ^2^ (66) =87.083, p=0.042 *
Diploma	χ ^2^ (8) =10.000, p=0.265	χ ^2^ (12) =15.000, p=0.241	χ ^2^ (12) =15.000, p=0.241
Family Type			
Joint Family	χ ^2^ (104) =235.627, p=0.000 *	χ ^2^ (96) =208.574, p=0.000 *	χ ^2^ (216) =470.522, p=0.000 *
Nuclear Family	χ ^2^ (35) =42.472, p=0.180	χ ^2^ (45) =51.897, p=0.223	χ ^2^ (48) =74.000, p=0.009 *
Occupation			
Homemaker	χ ^2^ (98) =180.349, p=0.000 *	χ ^2^ (91) =170.998, p=0.000 *	χ ^2^ (180) =344.503, p=0.000 *
Homemaker resigned	χ ^2^ (40) =48.889, p=0.158	χ ^2^ (15) =20.854, p=0.142	χ ^2^ (36) =44.000, p=0.169
Education Sector	χ ^2^ (6) =6.000, p=0.423	χ ^2^ (16) =21.333, p=0.166	χ ^2^ (15) =15.111, p=0.443
Others	χ ^2^ (35) =64.800, p=0.002 *	χ ^2^ (40) =45.667, p=0.248	χ ^2^ (24) =41.550, p=0.014 *

* Good level of significance (p < 0.05).

### Concerns of primiparous mothers towards newborn health and development

**Figure 5.**
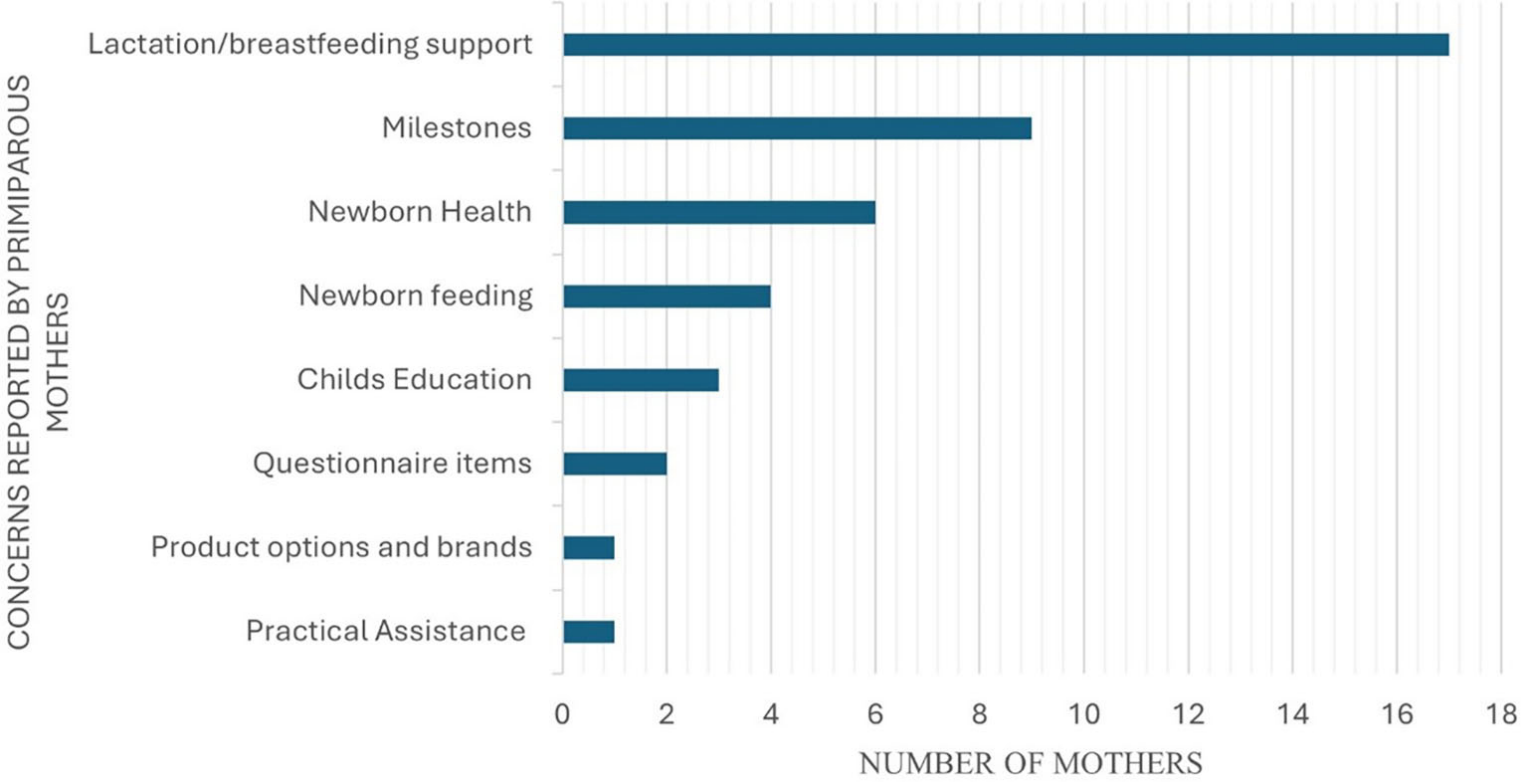
Concerns of primiparous mothers on newborn health and development.

Following the administration of the AQ (pre-test), a variety of responses were obtained from primiparous mothers through open-ended questions pertaining to concerns on newborn care and development as well as their perceived importance of communication with the newborn. Figure 5 represents the identified concerns of primiparous mothers about newborn development.

### Perspectives of primiparous mothers towards the importance of communication skills during infant development

A certain proportion of primiparous mothers (5%) reported that communication with their infants will (1) obtain better responses from the newborn, (2) facilitate in general and intellectual development of the newborn, (3) develop social skills, (4) encourage play behaviours in newborns, and (5) improve mother-child bonding. However, a few mothers (2%) reported that communication with their newborns may not necessarily be important in the early years but becomes crucial only when the child becomes older. Additionally, some mothers (3%) reported to be unaware of the importance of communication with the newborn.

### Feedback of primiparous mothers towards the N-CHFSEP

The results of the FQ revealed 67%, 32% and 1% of primiparous mothers to strongly agree, agree, and neutrally agree respectively towards the effectiveness of the education program. Following the administration of the FQ, a variety of responses were gathered from the open-ended question (Item no. 6). Several mothers (50%) reported that N-CHFSEP benefitted them by (1) providing additional information on communication skills, (2) offering a comprehensive view on the timelines of the developmental process, (3) emphasizing the importance of hearing, (4) addressing feeding concerns, and (5) providing insights into the newborn health. In addition, the programme was effective in (6) establishing the common disbelief that typically male children may verbally communicate much later than female children, and (7) understanding the behaviours to be observed during their newborn development. Others expressed a need for specific details pertaining to (8) fever management, (9) troubleshooting lactation failure and its effect on the baby, (10) details on avoidance of the type of feeds for the infant, and (11) developmental aspects beyond 6 months were reported by 8%, 15%, 5%, and 5% of the mothers respectively.

## Discussion

The result of the current study portrays the confidence levels of primiparous mothers before and after being subjected to the N-CHFSEP, and the possible influencing variables (age, education, family type, and occupation) on the same. Around 46%, 47%, and 61% of primiparous mothers reported heightened confidence levels in recognizing communication skills, feeding-swallowing skills, and newborn health following receiving the N-CHFSEP, while 54%, 53% and 39% did not report of any positive change in their confidence levels respectively. This observed change (pre and post) was statistically significant as per paired t-test analysis (p <0.05) (as reported in Table 2).

### Newborn communication skills

Considering the questions (in AQ) pertaining to communication skills, 89 (for Item 1) and 84 (for Item 2) primiparous mothers reported having a pre-existing understanding (pre-AQ administration) of these skills, attributing this to their exposure to a variety of informational sources (educational programs, books, social media platforms, etc.) which may have helped them prepare well in advance for judging their newborn communication skills. Australian researchers reported variability in the knowledge of primiparous mothers on early communication development to often rely on friends and social media for information, which was mostly unfortunately deemed unreliable according to maternal reports ( Williams et al., 2014). Catering to the perspectives of primiparous mothers towards the importance of communication skills in the current study, it was reported that communication with newborns helps them to obtain responses, facilitating in general and intellectual development of their newborn, developing social skills, encouraging play behaviours, and improving mother-child bonding. However, a few mothers reported that communication with their newborns may not necessarily be important in the early years but becomes crucial only when the child becomes older. Additionally, some mothers reported to be unaware of the importance of communication with the newborn. As per the concerns obtained (AQ-Item 18) from the participants of the current study, certain mothers reported to incorrectly estimate the developmental milestones in newborns which was in line with reports ( Huang et al., 2005). Several mothers also reported a lack of communication between healthcare professionals regarding newborn language development ( Suskind et al., 2018). However, the evident increase in the confidence levels (p<0.05) of the primiparous mothers in identifying the newborn communication skills in the current study (AQ-Item 1 and 2), can be attributed to the increased awareness these mothers would have received by the N-CHFSEP. As per the N-CHFSEP, the domains specified pertaining to communication development tapped upon milestones including the presence of a social smile, recognition of the mother, patterns of vocalization and babbling and communicative behaviours. The provision of such information may have imparted new knowledge on their newborn communication behaviours leading to increased confidence levels (p <0.05) as reported in Table 2. A randomized control trial done to study the effectiveness of educating parents on infant language development, parents were found to have enhanced knowledge about early language environments and caregiver responsiveness using a newborn program, in conjugation with a newborn hearing screening program ( Suskind et al., 2018).

Ninety-two mothers reported a heightened increase in the confidence levels in identifying their newborns hearing abilities (AQ-Item 3) prior to the education program. This could be attributed to their awareness of the existing policies and implementation of newborn hearing screening programs available in Indian hospitals ( Jatto et al., 2018) (including the one in which the participants of the current study were admitted for delivery). Following the N-CHFSEP delivery to the participants of the current study, an increase in their confidence levels (p <0.05) for Item 3 was observed. The N-CHFSEP items pertaining to this aspect (on hearing loss) were directed to the knowledge and understanding of the timeline when their baby would ‘appear to listen and pay attention to sounds’, ‘localization of the speaker’, and ‘recognizing and responding to their name call’. An increase in the early detection of hearing loss with a corresponding improvement in language skills, behaviours, and the quality of life of infants due to universal newborn hearing programs has been reported ( Edmond et al., 2022).

### Newborn feeding-swallowing skills

Considering the questions (in AQ) pertaining to feeding-swallowing skills, 89 (for Item 4) and 93 (for Item 12) primiparous mothers reported having a pre-existing understanding (pre-AQ administration) of these skills. This may be attributed to various factors such as (1) their personal experiences in observing feeding newborns within the family circle, (2) mothers prior to delivery may have self-educated themselves about feeding skills from other support networks, and (3) the availability of lactation counsellors in hospitals offering mothers with practical assistance and opportunity to discuss feeding related concerns during the hospital stay. This aligns with previous findings in India, which reported 40% of the mothers to have received antenatal education on breastfeeding practices, while 61% were educated by family and friends ( Sultania et al., 2019). The study also reported that most mothers had good knowledge about breastfeeding and the nutritional advantages of the same, while only 45% of the mothers practiced the knowledge they gained through these sources. As per the concerns obtained (AQ-Item 18) from the participants of the current study (as indicated in Figure 5), certain mothers reported concerns about breastfeeding knowledge and support. These included concerns towards deciding the feeding position, feeding duration, feeding schedule, feeding precautions, post-feeding management, aspiration management, and the type of feeds. The evident increase in the confidence levels (p<0.05) of the primiparous mothers (in the current study) in identifying newborn feeding schedules and understanding of exclusive breastfeeding for six months (AQ-Item 4 and 12), can be attributed to the adequate information provided in N-CHFSEP such as ‘exclusive breastfeeding’, ‘burping’, ‘feeding duration’ and ‘schedule’, and ‘awareness on the usage of formula feeds’. This provision of information likely complemented their existing knowledge of newborn feeding and swallowing skills. Moreover, practical demonstrations by lactation counsellors further reinforce their understanding, contributing to an increase in their confidence levels as reported in Table 2. This finding is consistent with the findings of a systematic review done on the effectiveness of educational programs related to breastfeeding and supportive programs for primiparous mothers. The studies reported the mothers to have increased breastfeeding knowledge and self-efficacy, thereby increasing the frequency of breastfeeding ( Wong et al., 2021).

### Newborn general health

Considering the questions (in AQ) pertaining to newborn health (AQ-Items 10, 11, 13 and 14), primiparous mothers reported having a pre-existing understanding (pre-AQ administration) of these skills. This may be attributed to the information provided on newborn hygiene and health status by the health professionals during their follow-up visits during the post-pregnancy/natal period. These heightened confidence levels may also be attributed to the significant role played by caregivers demonstrating and providing practical training on newborn care skills and the availability of educational cards such as Mother Child Protection Cards helping mothers to track newborn development, immunization schedules, and its importance. These findings were reaffirmed in another South India study, where 99.5 % of first-time mothers were aware of the importance of immunization for their children from various sources ( Kumar et al., 2015). Some concerns expressed by the participants of the current study (AQ-Item 18), were regarding their child’s future education, selection of high-quality products to ensure child safety, precautions towards general health, weight gain, and bathing care. Following the N-CHFSEP delivery to the participants of the current study, an increase in their confidence levels (p <0.05) for Items 10,11, 13 and 14 was pertaining to newborn health observed in the mothers. This could be attributed to the N-CHFSEP which included adequate information on identifying the appropriate vitamin supplements, newborn hygiene practices, and post-immunization effects. Though the mothers were confident and aware of these aspects for their newborns, the N-CHFSEP supplemented their knowledge by providing comprehensive information on the administration timeline and schedules on the same. This aligns with previous research indicating the effectiveness of flipchart-assisted maternal education in improving breastfeeding practices, hygiene skills, and temperature maintenance in newborns after the educational intervention ( Eluri et al., 2022). Considering the mothers confidence levels in independently identifying developmental milestones and precautions towards hypothermia (AQ-Items 15 and 16), there was an increase in the number of mothers (91 and 92 mothers respectively) exhibiting heightened confidence levels following the N-CHFSEP delivery, compared to AQ-Items 10, 11, 13 and 14. This could be attributed to the N-CHFSEP, which provided adequate information offered in the child-rearing practices such as monthly milestones, and suggestions to prevent hypothermia. In contrast to many educational programs that do not emphasize comprehensive monthly milestones, especially those that are not verbally presented to these mothers, the N-CHFSEP (in the current study) was provided via a one-to-one educational session, which was advocated by previous research as well ( Khresheh et al., 2011). The findings of the current study were consistent with a previous study done in the USA, where the researchers found a higher score in newborn care knowledge and maternal confidence after the provision of a newborn class program ( Gozali et al., 2020).

### Warning signs

There was an increase in the mother’s confidence levels post N-CHFSEP delivery in independently identifying the warning signs (AQ-Items 5 and 7) during the infant’s communication development. This may be attributed to the aspects of N-CHFSEP which included warning signs on ‘social smile’, ‘types of child response’, ‘attentional skills’, and ‘patterns of vocalizations’ along with the corresponding timelines, thereby helping them identify potential issues in newborn development. Previous studies have proclaimed a potential lack of awareness among caregivers on implementing communication strategies ( Skeat et al., 2010). The N-CHFSEP provided mothers with strategies to be used at home to increase communication skills in newborns, thereby increasing their confidence levels. Considering the questions (in AQ) pertaining to the warning signs (AQ-Items 8 and 9) in feeding-swallowing skills, there was a decrease in the number of mothers (77 and 81) reporting to be confident (before N-CHFSEP) compared to items 4 and 12 (89 and 93). This decrease may be attributed to various factors such as lack of previous experiences in newborn care, fear of misinterpretation of potential warning signals, increased stress levels during the early motherhood period, limited access to antenatal and postnatal services, and information overload during the post-pregnancy period. These factors may have led mothers to a state of confusion, making it difficult to distinguish and retain essential information related to newborn care feeding skills. This contradicts to the findings of an earlier study conducted in Punjab which reported 49.6% of urban mothers to have a good knowledge of newborn danger signs ( Singh et al., 2021). The current study mothers were provided with adequate information on neonatal danger signs such as ‘struggling to swallow’, ‘feeds coming out of the baby’s nose immediately after being fed’, ‘vomiting of a large amount of feeds’, ‘coughing immediately after feeds’, ‘baby appearing dull and unable to take feeds’, and ‘forehead sweating immediately after feeds’. This was in line with a previous South Indian study, which included newborn feeding and health danger signs ( Thomas & Devi, 2020). The study demonstrated the effectiveness of a structured teaching program in improving the knowledge of postnatal mothers on newborn danger signs including those related to feeding and newborn health.

### Factors influencing mother’s confidence levels

The factors influencing the confidence levels of primiparous mothers (in the current study) could be attributed to the timing of intervention (provided second day of post-delivery), the expertise and the training provided by the health care professionals (SLP and Paediatrician), and the structure of the provided education program (monthly milestones, suggestions and danger signs), all of which being reiterated in previous studies ( Ben-Sasson et al., 2022). Most participants in the current study were recruited from Government Lady Goschen Hospital, Mangalore which is exclusive for maternity care, encompassing a team of health care professionals from various disciplines. Studies have emphasized the significance of healthcare professionals as the primary source of information when queried about the benefits of postpartum educational programs ( Gozali et al., 2020). Additionally, the hospital (in the current study) offered several instructional resources in Kannada and English such as public awareness posters on early newborn care skills and Mother Child Protection cards supplementing their confidence levels.

The age, education, occupation, and family type of the participants (in the current study) had a significant effect (p<0.05) on the confidence levels pre and post N-CHFSEP administration. The maternal age (18-25 and 26-35 years) presented an effect (p <0.05) on the confidence levels in communication skills, feeding-swallowing skills, and newborn health as reported in Table 3. The higher confidence levels observed among both young and older mothers could be attributed to their inclination to use social media and mobile applications ( Ben-Sasson et al., 2022), which are generally free and readily available sources of information about newborn care. Such applications enable mothers to get information on monthly milestones, paediatric weight gain, vaccination charts, reminders on health checkup days, etc. Access to such information could contribute to increased confidence levels in managing various domains of communication skills, feeding-swallowing skills, and newborn health. Applications such as the Baby Buddy App provided easily accessible and reliable pregnancy information aiding first time mothers to effectively communicate with healthcare professionals ( Bailey et al., 2022).

Maternal education levels presented a significant effect (p <0.05) on the confidence levels pre and post N-CHFSEP administration as reported in Table 3. The higher educational levels (Degree, <=10 ^th^ Std, <=12 ^th^ Std) were observed to have an effect (p <0.05) on the confidence levels of newborn communication skills and general health whereas the lower education level (<=10 ^th^ standard) specifically presented a significant effect (p <0.05) on the confidence levels in feeding-swallowing skills. This could be attributed to a deeper understanding of newborn care among highly educated mothers because of their formal education and exposure to various informational resources. Researchers found mothers with no formal education to score less than those who had higher education ( Memon et al., 2019). Higher levels of education may cause an urge among these mothers to obtain evidence-based resources from reliable websites and scientific journals. This inclination may prompt them to seek clarification from qualified healthcare professionals when needed.

According to the results reported in Table 3, a significant effect (p <0.05) on maternal confidence levels on overall newborn care and development was observed among those who belonged to a joint and a nuclear family type. With a joint family structure generally comprising grandparents (experienced caregivers) or extended families, it becomes natural for first-time mothers to absorb traditional methods of newborn care along with practical demonstrations, thereby boosting their confidence levels. Regular interaction and discussions by these caregivers provide mothers with practical suggestions and solutions in various aspects of newborn care. The joint family type facilitates obtaining collective information on effective parenting strategies and decision-making during certain newborn practices such as feeding, hygiene practices, and newborn health practices, reassuring the first-time mothers in the process. Surprisingly, first time mothers from nuclear families, undertake greater responsibilities towards newborn care even without the presence of extended family members. These mothers get trained themselves to become competent in newborn care practices. The provision of N-CHFSEP in the current study may have addressed these maternal concerns by equipping them with monthly milestones, newborn care suggestions, and monthly warning signs, thereby causing a positive effect on their confidence levels.

Maternal occupations such as homemakers and others (government and private officials) were found to have a significant effect (p<0.05) on the confidence levels towards communication and feeding-swallowing skills as reported in Table 3. However studies have not found any significant association between mothers’ occupation and newborn care ( Memon et al., 2019; Tegene et al., 2015). Homemakers generally get opportunities to engage in interactions with their newborns thereby fostering newborn language development and communication skills. Their consistent presence at home provides opportunities for monitoring newborn care and interventions when required. Although being a homemaker is highly advantageous, there do exist challenges such as feeling isolated, lacking professional advice, and feeling financial constraints, making them less confident in newborn practice in the process. Access to resources such as government-sponsored healthcare programs or childcare services, could positively impact their ability to prioritize development of feeding skills. The provision of N-CHFSEP might have complemented and enhanced the caregiving practices of both homemakers and government officials.

### The path forward

With the implementation of N-CHFSEP in the current study, the primiparous mothers received a comprehensive education on various aspects of newborn skill development. The observed increase in confidence levels following N-CHFSEP suggested the program to effectively complement the mother’s knowledge and understanding, thereby contributing to improved confidence and awareness regarding newborn care. This understanding is crucial for the implementation of comprehensive educational interventions addressing the specific needs of primiparous mothers as it may lead to enhanced maternal and infant health outcomes. As per the obtained feedback (FQ-Item 6) from the current study participants, the N-CHFSEP provided mothers with additional information in various aspects regarding communication skills, emphasizing the importance of hearing, and corrected misconceptions. Studies have found feedback of educational programs to have positive outcomes such as encouraging mothers to interact and read with their infants in a responsive manner. These mothers reported being willing to recommend the education program to others and apply the knowledge with their newborns at home ( Suskind et al., 2018). A study on the NewBorn Class program reported that it benefitted approximately 58% of the mothers by providing comprehensive knowledge for first-time mothers, and providing information on medical topics such as feeding, diaper rash, and developmental skills ( Gozali et al., 2020). A total of 38 (39%) mothers provided a feedback on the N-CHFSEP program. Although certain mothers claimed to be already aware of the developmental expectations of the newborn (from the MCP card they received from the hospital), others demanded additional information on fever management, troubleshooting lactation failure and its effect on the baby, details on avoidance of the type of feeds for the infant, and developmental aspects up to 1 year of infant’s age (for which they were willing to have an additional consultation post 6 months). Although some mothers reported to lack the awareness of monthly milestones and felt boys spoke later than girls, many admitted they now know the importance of communication and hearing. Furthermore, some mothers highlighted the need for hands-on application of the information provided, thereby suggesting N-CHFSEP to be supplemented with practical demonstrations to enhance their confidence levels. Although the developed N-CHFSEP showed a significant positive change (pre and post-administration) in the attitude levels of the mothers, having a control group (mothers without receiving the intervention program) and comparing them to an experimental group (mothers receiving the intervention program) would have increased the validity of the tool. The current study findings cannot be generalized to multiparous mothers and mothers from diverse cultural or family backgrounds. Furthermore, the effectiveness of N-CHFSEP was determined immediately after the administration, leaving uncertainty about information retention up to 6 months post-intervention. The N-CHFSEP included information on neonatal care from only two disciplines (SLP and Pediatrics), suggesting the possible inclusion of additional members (such as fathers, grandparents, lactation counsellors, psychologists, Accredited Social Health Activist workers, nurses, etc.) to provide a more comprehensive understanding of various aspects of newborn health and development. Since the current work is a preliminary study, it would therefore be interesting if future studies would consider controlling the above-mentioned factors (and feedback from mothers) with a larger sample size to further derive a highly valid educational program.

## Ethics and consent

The study was approved by the Institutional Ethics Committee (IEC KMC MLR 03/2023/108) of Kasturba Medical College, Mangalore, Manipal Academy of Higher Education on 20.07.2023 and was registered under the Clinical Trials Registry of India (CTRI/2023/05/053109) on 25.05.2023. The researcher obtained written informed consent from the participants followed by their socio-demographic details.

## Data Availability

*Name of the repository*: Mendeley Data *Project title*: Estimating the efficacy of Newborn-Communication, Health, Feeding and Swallowing Education Program (N-CHFSEP) for primiparous mothers. https://data.mendeley.com/datasets/7c75ws94vn/2 ( Ouseph et al., 2024). This project contains the following underlying data:
• DATA ENTRY SHEET.xlsx (includes data on the patient demographics, responses from pretest Attitude Questionnaire, posttest Attitude Questionnaire, and Feedback Questionnaire) • Read Me.txt (description to understand the variables in data files) •
completed_CONSORT_checklist.docx (includes the CONSORT checklist)
*License*: Creative Commons Attribution 4.0 International license (CC-BY 4.0).
