# Supplementary material for: Evaluating the Efficacy of the Newborn Communication, Health, Feeding, and Swallowing Education Program (N-CHFSEP) for First-Time Mothers in Mangalore taluk, Dakshina Kannada district, Karnataka, India: A Preliminary Study
Source: F1000Res. 2025 Jan 31;13:777. Originally published 2024 Jul 9. [Version 2] doi: 10.12688/f1000research.152320.2 (PMC11862371; doi:10.12688/f1000research.152320.2)
Supplement: Peer review history

# Reviewer report: Reviewer response for version 2

**DOI:** 10.5256/f1000research.176318.r367189

**Published:** 2025-02-25

**Authors:** Lucia Lombardi

**Affiliations:** 1 University "G. d'Annunzio", Chieti-Pescara, Chieti, Italy

This study aimed to assess the efficacy of an educational program designed to improve primiparous mothers’ attitudes toward newborn communication, general health, feeding, and swallowing. 

 Authors described two phases: 1) developed ad hoc questionnaires, 2) administration of these tools.

 The article is very interesting, clear and well written. I suggest accept this article. 

 Kind regards.

Is the work clearly and accurately presented and does it cite the current literature?

Yes

If applicable, is the statistical analysis and its interpretation appropriate?

Yes

Are all the source data underlying the results available to ensure full reproducibility?

Yes

Is the study design appropriate and is the work technically sound?

Yes

Are the conclusions drawn adequately supported by the results?

Yes

Are sufficient details of methods and analysis provided to allow replication by others?

Yes

Reviewer Expertise:

At risk parentinggenetic counseling

I confirm that I have read this submission and believe that I have an appropriate level of expertise to confirm that it is of an acceptable scientific standard.

# Reviewer report: Reviewer response for version 2

**DOI:** 10.5256/f1000research.176318.r363723

**Published:** 2025-02-17

**Authors:** Amogh Verma

**Affiliations:** 1 Rama Medical College Hospital and Research Centre,, Hapur,, India

I have reviewed the revised version of the manuscript, and I confirm that the authors have acted upon all my comments. I have no further concerns at this stage.

Is the work clearly and accurately presented and does it cite the current literature?

Yes

If applicable, is the statistical analysis and its interpretation appropriate?

I cannot comment. A qualified statistician is required.

Are all the source data underlying the results available to ensure full reproducibility?

Yes

Is the study design appropriate and is the work technically sound?

Yes

Are the conclusions drawn adequately supported by the results?

Yes

Are sufficient details of methods and analysis provided to allow replication by others?

Yes

Reviewer Expertise:

NA

I confirm that I have read this submission and believe that I have an appropriate level of expertise to confirm that it is of an acceptable scientific standard.

# Reviewer report: Reviewer response for version 1

**DOI:** 10.5256/f1000research.167068.r318054

**Published:** 2024-09-03

**Authors:** Amogh Verma

**Affiliations:** 1 Rama Medical College Hospital and Research Centre,, Hapur,, India

This study evaluates the effectiveness of a tailored educational program, the Newborn-Communication, Health, Feeding, and Swallowing Education Program (N-CHFSEP), in enhancing the confidence of primiparous mothers in newborn care. The research addresses a significant gap in maternal education, particularly in the context of first-time mothers who face unique challenges in caring for their newborns.

**Strengths**:

1. **Relevance and Impact**: The study addresses a highly relevant topic in maternal and child health. The focus on primiparous mothers and the development of a comprehensive educational program is commendable, particularly in regions where such resources may be limited.
2. **Methodological Rigor**: The development and validation of the study tools (Attitude Questionnaire, Feedback Questionnaire, and N-CHFSEP) are well-detailed and supported by content validation from experts in pediatrics and speech-language pathology.
3. **Statistically Significant Findings**: The study presents statistically significant improvements in maternal confidence levels across communication, feeding-swallowing, and general health domains, which suggests that the N-CHFSEP is an effective intervention.
4. **Practical Implications**: The study provides valuable insights for healthcare providers and policymakers, highlighting the importance of structured educational programs for new mothers.

**Weaknesses**:

1. **Study Design**: The absence of a control group limits the ability to attribute the observed improvements in confidence levels solely to the N-CHFSEP intervention. This is a significant limitation that should be addressed in future studies. The single-arm pre-post study design, while valid for exploratory research, does not provide the level of rigor necessary to establish causality.
2. **Generalizability**: The sample is limited to primiparous mothers in a specific region, and the exclusion of multiparous mothers may limit the generalizability of the findings to the broader population. Expanding the sample to include a more diverse demographic would strengthen the study.
3. **Short-term Assessment**: The study measures outcomes immediately post-intervention, leaving questions about the long-term retention of knowledge and skills. A follow-up assessment at 6 months or beyond would provide a more comprehensive understanding of the program's sustained impact.
4. **Limited Qualitative Data**: While quantitative data is well-represented, the qualitative feedback from participants is not fully explored. Incorporating more qualitative insights could provide a richer context to the statistical findings and highlight areas for improvement in the program.

**Recommendations for Publication**:

- **Revisions**: I recommend that the authors address the limitations in their discussion section by clearly acknowledging the absence of a control group and the implications for the study's findings. Additionally, suggestions for future research should be included, particularly regarding long-term follow-up and expanding the sample population.
- **Potential for Improvement**: The study would benefit from a more in-depth analysis of the qualitative data collected, as this could provide valuable insights into the participants' experiences and the practical application of the program. Additionally, including recommendations for enhancing the program, such as integrating practical demonstrations, would be beneficial.
- **Suitability for Indexing**: Despite its limitations, the study contributes valuable insights into maternal education and has practical implications for improving maternal and infant health. I believe the manuscript is suitable for indexing with revisions. However, the authors should emphasize that this is a preliminary study, laying the groundwork for more rigorous future research.

Is the work clearly and accurately presented and does it cite the current literature?

Yes

If applicable, is the statistical analysis and its interpretation appropriate?

I cannot comment. A qualified statistician is required.

Are all the source data underlying the results available to ensure full reproducibility?

Yes

Is the study design appropriate and is the work technically sound?

Yes

Are the conclusions drawn adequately supported by the results?

Yes

Are sufficient details of methods and analysis provided to allow replication by others?

Yes

Reviewer Expertise:

Internal Medicine, Pediatrics, Allergy and Immunology, Surgery and Emergency Medicine.

I confirm that I have read this submission and believe that I have an appropriate level of expertise to confirm that it is of an acceptable scientific standard, however I have significant reservations, as outlined above.

# Reviewer report: Reviewer response for version 1

**DOI:** 10.5256/f1000research.167068.r303850

**Published:** 2024-07-29

**Authors:** shaimaa Mohamed Amin

**Affiliations:** 1 Damanhur University, Damanhour, Egypt

**Dear [editor ],**

 I hope this message finds you well. I wanted to extend my sincere gratitude to you for sending me the article for review. I truly appreciate the opportunity to contribute to the process and offer my insights.

 I've gone through the article and I have some constructive feedback that I believe could enhance the overall quality and impact of the piece.

- **Title: **Your research title is clear and informative, but it can be made more concise and engaging. Here are a few suggestions to refine and enhance the title:
- Evaluating the Efficacy of the Newborn Communication, Health, Feeding, and Swallowing Education Program (N-CHFSEP) for First-Time Mothers

- Assessing the Impact of the N-CHFSEP on Newborn Care Among Primiparous Mothers
- Effectiveness of the N-CHFSEP in Enhancing Newborn Care Skills for Primiparous Mothers

- **Abstract **

- **Background:** The background section effectively outlines the problem that primiparous mothers face challenges during pregnancy and post-childbirth. However, the statement "There is a lack of comprehensive educational programs for primiparous mothers on maternal functioning and newborn care" could benefit from citation of existing literature to support this claim. Additionally, specifying what aspects of "maternal functioning" are included could provide clarity.
- **Objectives:** The objectives are clearly stated and logically divided into two main goals: development of tools (attitude questionnaire, education program, feedback questionnaire) and evaluation of the program's efficacy. This separation is clear and helpful for readers to understand the study’s aims.
- **Methods:** The methods section is concise but detailed enough to understand the study design. However, it could be improved by specifying the inclusion criteria more precisely (e.g., "primiparous mothers without any obstetric history" could specify what kind of history excludes participants). Additionally, mentioning the duration of the study and the specific content covered in the N-CHFSEP could provide more context.
- **Results:** The results section effectively communicates the main findings, highlighting the statistically significant increase in maternal confidence levels post-intervention. The use of both quantitative and qualitative analysis is a strength. However, the phrase "a notable increase in the number of mothers (not all)" is vague and could be more precise. For example, specifying the percentage of mothers who reported increased confidence would provide more concrete data. Additionally, discussing the specific sociodemographic factors in more detail would enhance understanding of their impact.
- **Conclusions:** The conclusions succinctly summarize the study's implications, emphasizing the program's effectiveness in enhancing maternal confidence. However, it would be beneficial to briefly mention any limitations of the study or suggest directions for future research to provide a more balanced view.
- **Keywords:** The keywords are relevant and cover the main topics of the study. However, adding keywords like "confidence," "maternal education," and "program evaluation" might improve the searchability of the study.

- **Introduction: **The introduction provides a comprehensive overview of the challenges faced by primiparous mothers and underscores the importance of educational programs to support them. Here are some suggestions to refine and strengthen the introduction:

1. **Clarity and Focus:**
  - The introduction covers a broad range of issues and studies, which can make it somewhat dense. Consider focusing more sharply on the main problem and the gap your study aims to fill. For example:
    - Highlight the specific challenges primiparous mothers face and how these impact newborn care.
    - Clearly state the need for a comprehensive educational program that addresses these challenges.
2. **Structure:**
  - **First Paragraph:** Introduce the general context of pregnancy and childbirth, emphasizing the unique challenges for primiparous mothers.
  - **Second Paragraph:** Discuss the importance of maternal confidence, knowledge, and attitudes, and their impact on newborn care.
  - **Third Paragraph:** Present the specific gaps in current educational programs, citing key studies that demonstrate the need for comprehensive support.
  - **Fourth Paragraph:** Highlight existing educational programs and their limitations, particularly focusing on the need for a holistic approach.
  - **Fifth Paragraph:** Conclude by summarizing the need for your study and its objectives.
3. **Citations and Evidence:**
  - Ensure all claims are supported by citations. For example, when discussing the impact of maternal confidence or the effectiveness of various programs, provide specific references.
  - Use consistent and current references to strengthen the credibility of your argument.
4. **Flow and Readability:**
  - Improve readability by breaking long sentences into shorter, more concise ones.
  - Use transition phrases to connect ideas and ensure a smooth flow from one paragraph to the next.
5. **Specific Suggestions:**
  - **Opening Sentence:** "Pregnancy and childbirth represent significant milestones in a woman's life, permanently altering her identity and way of living in a continuous and dynamic manner."
  - **Second Sentence:** "Primiparous mothers (first-time mothers) face a wide range of emotions including joy, excitement, and anxiety, alongside overwhelming and stressful experiences such as routine newborn care, breastfeeding difficulties, lack of sleep, and physically taxing household duties."
  - **Importance of Maternal Confidence:** "Reduced levels of confidence in primiparous mothers compared to multiparous mothers negatively impact their ability to provide infant care."
  - **Developmental Milestones:** "Effective identification of developmental milestones by caregivers facilitates early interventions, improving overall health outcomes."
  - **Educational Programs:** "Although numerous educational programs exist, there is a lack of a holistic approach that comprehensively addresses newborn communication, feeding, swallowing, and general health."
6. **Conclusion of Introduction:**
  - **Summarize Gaps and Objectives:** "Despite the availability of various educational initiatives, there is a noticeable gap in comprehensive programs that address all critical areas of newborn development. This study aims to develop and validate a comprehensive educational program and assess its efficacy in enhancing maternal confidence and knowledge among primiparous mothers."

- **Methods **

**Study Design and Ethics:**

- **Clarity:** This section is clear and provides essential information about the study design and ethical approvals.
- **Detail:** Including the registration number and ethical approval details adds credibility. Mentioning the adherence to the CONSORT checklist and Declaration of Helsinki is crucial.

**Participants:**

- **Clarity:** The paragraph provides detailed demographic data which is good for understanding the sample population.
- **Structure:** Breaking this into two paragraphs might enhance readability - one for sample size calculation and the other for demographic details.
- **Detail:** Including the sample size formula and demographic breakdown is thorough and helpful.

**Inclusion and Exclusion Criteria:**

- **Clarity:** The criteria are clearly listed, which helps in understanding the participant selection process.
- **Structure:** The criteria are clearly separated into inclusion and exclusion, making it easy to follow.
- **Detail:** Including the proficiency in English or Kannada is important for understanding participant communication abilities.

**Procedure: **The present study was conducted in 2 phases. Phase 1 included the development of an (a) attitude questionnaire, (b) parent education program, and (3) feedback questionnaire; while Phase 2 included the administration of the questionnaires and the education program on the participants, followed by data analysis of the retrieved data.

- **Clarity:** The procedure is outlined, indicating a clear structure to the study.
- **Detail:** Describing the phases helps in understanding the study's flow.

**Development of Tools:**

 a) Attitude questionnaire (AQ):

- **Clarity:** The development process of the AQ is well-explained, detailing the domains and types of questions.
- **Detail:** Including specific item numbers and their domains adds precision.

b) Parent Education Program

 : **Clarity:** The development process of the N-CHFSEP is described in detail.

- **Detail:** Mentioning the consultation with experts adds credibility.

c) Feedback Questionnaire (FQ):

**Clarity:** The development process of the FQ is clear and detailed.

- **Detail:** Including the types of questions adds precision.
- **Discussion **

The discussion section of this study provides a comprehensive analysis of the impact of the Newborn Communication, Hearing, Feeding, and Swallowing Education Program (N-CHFSEP) on the confidence levels of primiparous mothers, emphasizing key areas such as communication, feeding-swallowing skills, and newborn health. While the study effectively highlights the statistical significance of increased confidence post-intervention and relates these findings to previous research, it could benefit from a more concise presentation. The detailed breakdown of influencing variables (age, education, family type, and occupation) is insightful, yet the narrative occasionally becomes repetitive, potentially diluting the focus. Additionally, the discussion extensively references existing literature to contextualize findings, which is commendable, but a more balanced approach with critical reflections on the study's limitations, such as the lack of a control group and the short-term assessment of the intervention's impact, would enhance the overall analysis. The feedback from mothers and the suggestion for practical demonstrations underscore the need for a hands-on approach in educational programs, a point that could be more prominently integrated into the discussion. Overall, while the discussion is thorough and well-supported by data, a more streamlined and critically reflective narrative would strengthen its impact

- **Please address conclusion,  limitations & implications  of the study **

**Once again, thank you for entrusting me with this task. I look forward to our continued collaboration and to seeing the final version of the article.**

**Warm regards,**

 Shaimaa Mohamed Amin

Is the work clearly and accurately presented and does it cite the current literature?

Yes

If applicable, is the statistical analysis and its interpretation appropriate?

Partly

Are all the source data underlying the results available to ensure full reproducibility?

Yes

Is the study design appropriate and is the work technically sound?

Yes

Are the conclusions drawn adequately supported by the results?

No

Are sufficient details of methods and analysis provided to allow replication by others?

Partly

Reviewer Expertise:

community health nursing

I confirm that I have read this submission and believe that I have an appropriate level of expertise to confirm that it is of an acceptable scientific standard, however I have significant reservations, as outlined above.
